# Dual drug-loaded nano-platform for targeted cancer therapy: toward clinical therapeutic efficacy of multifunctionality

**DOI:** 10.1186/s12951-020-00681-8

**Published:** 2020-09-04

**Authors:** Zhe Ma, Nan Li, Bing Zhang, YuYu Hui, Ying Zhang, Peng Lu, Jiaxin Pi, Zhidong Liu

**Affiliations:** 1 State Key Laboratory of Component-based Chinese Medicine, Tianjin University of Traditional Chinese Medicine, Tianjin, 301617 China; 2 Engineering Research Center of Modern Chinese Medicine Discovery and Preparation Technique, Ministry of Education, Tianjin University of Traditional Chinese Medicine, Tianjin, 301617 China; 3 Institute of Traditional Chinese Medicine, Tianjin University of Traditional Chinese Medicine, Tianjin, 301617 China

**Keywords:** Targeted cancer therapy, Multidrug resistance, Synergistic anti-cancer, Nano-combination therapy, Metastasis

## Abstract

**Background:**

Poor targeting and penetration of chemotherapy drugs in solid tumors, and the development of resistance to chemotherapeutic agents are currently hindering the therapy of breast cancer; meanwhile, breast cancer metastasis is one of the leading causes of death in breast cancer patients. With the development of nanotechnology, nanomaterials have been widely used in tumor therapy.

**Results:**

A multi-functional nano-platform containing gambogic acid (GA) and paclitaxel (PTX) was characterized by a small size, high encapsulation efficiency, slow release, long systemic circulation time in vivo, showed good targeting and penetrability to tumor tissues and tumor cells, and exhibited higher anti-tumor effect and lower systemic toxicity in BALB/c mice bearing 4T1 tumor. GA not only overcame the multidrug resistance of PTX by inhibiting P-glycoprotein (P-gp) activity in MCF-7/ADR cells, but also inhibited MDA-MB-231 cells migration and invasion, playing a crucial role in preventing and treating the lung metastasis of breast cancer caused by PTX; meanwhile, the synergistic anti-tumor effect of GA and PTX has also been verified in vitro and in vivo experiments.

**Conclusion:**

Our data described the better recognition and penetration of tumor cells of R9dGR-modified versatile nanosystems containing GA and PTX, which exerted one stone three birds clinical therapeutic efficacy of multifunctionality.
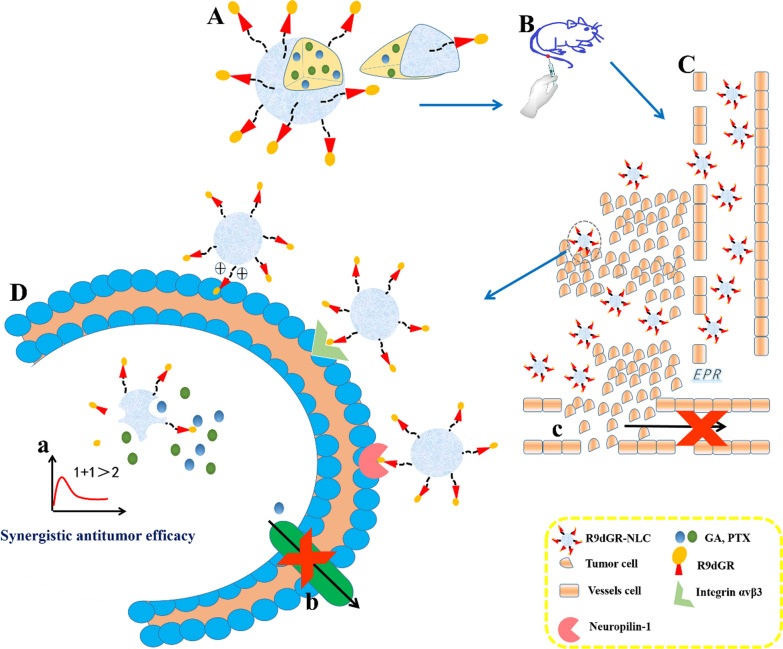

## Background

Cancer, as a malignant disease, is one of the leading causes of death in the world [1, 2]. Breast cancer, with the highest morbidity and mortality among various tumor types in women, is one of the malignant tumors [2, 3]. The poor targeting and permeability of chemotherapy drugs in solid tumors hinders the treatment of breast cancer.

Nanostructured lipid carriers (NLCs) are the second generation of novel lipid nanocarrier system after the traditional nanocarrier (nanoemulsion, liposome, and so on) and solid lipid nanoparticle (SLN), exhibiting better biocompatibility compared with polymer-based nano-carriers. NLCs, as mixtures of solid and liquid lipids, are characterized by higher encapsulation efficiency, excellent drug-loading capacity, better stabilization, sustained release properties, and the long resident time in blood [4]. Therefore, NLCs have dominant position in the treatment of diseases in special fields, including cancer [5].

An active drug delivery system targeting tumor has been developed to ensure that drugs induce the off-target effect in normal tissues so as to reduce damage to other normal cells by only acting on tumor cells [6]. The use of tumor-targeting and/or cell-penetrating peptides for functionalization of nanocarriers is a promising strategy, which has attracted researchers’ attention. The efficiency of polyarginine chain cell penetrating peptide (CPP) R9 with overwhelming cell penetration and internalization was 20 times higher than that of TAT peptide at penetrating the plasma membrane [7]. Based on this, a dual receptor targeted CPP with a sequence of RRRRRRRRRdGR-R9dGR was designed, which could bind to both integrin αvβ3 and neuropilin-1 (NRP-1) receptors that were high-expressed on breast cancer cells [8–10], and also penetrate tumor tissue by electrostatic interaction to realize the synergetic penetrable effect via the above three pathways. Moreover, RGERPPR (RGE, our previous results confirmed predominant efficiency of RGE in improving the targeting and penetrability of nanocarriers), having a high affinity toward NRP-1 receptor [9], was added into the study as an efficient CPP.

The combined therapy exhibits a crucial role for cancer treatment in synergy and complementarity. Its combination involves multiple targets and multiple signaling pathways, having an improved efficacy compared with single drugs [11].

Paclitaxel (PTX) is widely used in the clinical chemotherapy of breast cancer, ovarian cancer and other cancers. As a classical microtubule inhibitor, it is able to promote G2/M phase arrest and mitotic cell death [12, 13], and its curative effect is highly commended [14]. However, Taxol^®^ (PTX solution) easily causes nephrotoxicity, neurotoxicity and severe hypersensitivity reactions (HSRs) in patients attributed to the presence of cremophor [15, 16], in addition the clinical application of it is severely impaired by its short-term physical stability, short maintenance time of blood drug concentration, low concentration in tumor tissues, high incidence of cardiotoxicity, and poor targeting [17, 18]. While albumin-bound PTX nanoparticles (Abraxane^®^) still shows the rapid elimination of PTX from the blood circulation, which does not improve the in vivo pharmacokinetics of PTX compared to Taxol^®^ [19]. Meanwhile, as with other chemotherapeutic drugs, the emergence of PTX resistance is also a bottleneck restricting its clinical application [20]. While there is lacking supportive evidence that Abraxane^®^ is active against multidrug resistance (MDR) cancer. The mechanisms of PTX resistance in malignant tumors are extremely complex, but that over expression and abnormal function of drug resistance related proteins (P-gp, and so on) leading to the imbalance of PTX uptake and outflow, thus causing a decrease in the concentration of drugs in cells is an essential factor in the acquisition of PTX resistance [21]. Studies have confirmed that PTX has an ability to change the tumor microenvironment of metastasis (TMEM) and promote distant metastasis of breast cancer, increasing the risk of metastatic dissemination [22], which not only helps cancer cells escape from the primary focus, but also acts directly on the lungs, changing the microenvironment of the lungs and helping breast cancer cells colonize the lungs [23]. Therefore, as a first-line anti-tumor drug, the study of PTX to improve its water solubility, in vivo stability and targeting, reduce the multi-drug resistance and toxic side effects has been the difficulty and focus.

Gambogic acid (GA), a caged xanthone, is one of the effective ingredients of Gamboge that is a dry resin secreted by *Garcinia hanbaryi* Hook.F [24]. It was found that GA has the effects of anti-proliferation, induction of apoptosis and cell cycle arrest, possessing telomerase inhibition, antiangiogenic properties on certain tumors, such as gastric, lung, breast, pancreatic and prostate cancer cells [25–29]. Interestingly, compared with normal cells, GA can selectively induce cancer cell apoptosis, suggesting that GA may be a drug that is low toxic to normal cells but can effectively against cancer [30, 31]. And it has been approved by the National Medical Products Administration of China for phase II clinical trials [32]. In addition, Wang et al. found that GA could overcome drug resistant in breast cancer via inhibiting both P-gp activity and expression [26], which can reduce the efflux of P-gp substrates (including PTX) [33]. Moreover, GA could inhibit adhesion, migration, and invasion of cancer cells in vitro [34], which has provided the basis for GA as a potential drug for the therapy of breast cancer metastasis. GA has excellent antitumor activity, but its low solubility, short half-life and poor tumor selectivity hinder its clinical application [35].

The present study investigated the synergistic antitumor effect of PTX and GA, the properties of GA overcoming PTX resistance properties and suppressing lung metastasis of breast cancer. The combination therapy of GA and PTX could achieve the therapeutic effect of 1 + 1 > 2, and a clinical multifunctionality [36]. Meanwhile, RGE with predominant efficiency in improving the targeting and penetrability of nanocarriers and the first synthetic R9dGR were comprehensively evaluated to provide a few basic considerations for the application of CPP.

## Materials and methods

### Materials, cell lines and animals

GA with purity over 98% was obtained from Chengdu Herbpurify CO.,LTD (Chengdu, China); PTX with purity over 98% was obtained from Shanghai yuanye Bio-Technology Co., Ltd (Shanghai, China); Taxol^®^ (30 mg/5 mL) was purchased from Hospira Australia Pty Ltd; Myrj 52 (Polyethylene Glycol (40) Monostearate) and Compritol 888 ATO (Glycerol behenate) were purchased from Saint-Priest Cedex, France; LIPOID S-100 (Soybean Lecithin) was purchased from Shanghai Dong Shang Industrial Co., Ltd, China; MCT 812 (Miglyol^®^ 812) was obtained from Beijing Feng Li Jing Qi Trading Co., Ltd. China; DSPE-PEG_2K_-RGERPPR and DSPE-PEG_2K_-R9dGR peptides (Additional file 1: Figure S1, A: DSPE-PEG_2K_-COOH MALDI-TOF REPORT; B: RGERPPR MALDI-TOF REPORT; C: DSPE-PEG_2K_-RGERPPR MALDI-TOF REPORT; D: R9dGR(RRRRRRRRR-dGR) MALDI-TOF REPORT; E: DSPE-PEG_2K_-R9dGR (RRRRRRRRR-dGR) MALDI-TOF REPORT) were synthesized by Chinapeptide Biotech Co. Ltd. (Shanghai, China); 1,10-Dioctadecyl-3,3,3,3-tetramethyl indotricarbocyanine iodide (Dir) was purchased from Biotium Inc. (Hayward, CA); Coumarin-6 was obtained from Sigma (Saint Louis, MO); double antibiotics (10000U Penicillin streptomycin), 0.25% trypsin + 0.02% EDTA, and fetal Bovine Serum FBS were obtained from Gibco; Hematoxylin was purchased from Wuhan Biotechnology Co., Ltd (Wuhan, China); Cell Counting Kit 8 (CCK-8) was acquired from 758 R. HUANG ET AL. Dojindo Laboratories (Kumamoto, Japan); 96-well white basal cell culture plate, RMPI-1640 medium, and phosphate-buffered saline (PBS) were purchased from Corning, NY; Hoechst 33342 fluorescent probes were purchased from Sigma-Aldrich (MO, USA); aspartate aminotransferase (AST), serum creatinine (CRE), alanine aminotransferase (ALT), and creatine kinase (CK) detection assay kits were purchased from Nanjing Jiancheng Bioengineering Institute (Nanjing, China); All the other chemicals were analytical reagent grades, purchased from Tianjin Guangfu Fine Chemical Research Institute (Tianjin, China).

Human breast carcinoma MCF7, MDA-MB-231, and mice breast cancer 4T1 cells were acquired from ATCC (Manassas, VA). Human breast carcinoma MCF7/ADR was acquired from the Type Culture Collection of the Chinese Academy of Sciences (Shanghai, China). These tumor cells were cultivated in RPMI-1640 medium (89%, Corning) added to 10% FBS and 1% 100 IU mL^−1^ penicillin–streptomycin at 37 °C in a humidified atmosphere containing 5% CO_2_.

BALB/c nude mice (female, 6–8 weeks, 18–22 g) and BALB/c mice (female, 6–8 weeks, 16–19 g) were acquired from the Institute of Radiation Medicine Chinese Academy of Medical Sciences (Tianjin, China). Food and water are freely available for mice in a specific pathogen-free (SPF) environment at 25 ± 1 °C. White female rabbits (normal grade, New Zealand strain, weight 2.0 ± 0.5 kg) were acquired from Beijing huafukang biotechnology co., LTD. After a week of acclimation, these animals were used for experiments. All animal experiments were conducted in accordance with protocols, which was evaluated and approved by the Ethics Committee of Tianjin University of Traditional Chinese Medicine.

### Determination of combination index

The cytotoxicity of GA and PTX on 4T1 cells were measured by CCK-8 kit. The 4T1 cells were inoculated on a 96-well culture plate, and cultured for 24 h. The former medium in each well was replaced with 100 μl of culture medium containing different concentrations of either single drugs or drug combinations (GA, PTX). After 24 h, the culture medium containing drugs was removed, and 100 μL of 10% CCK-8 diluent were added into each well and incubated at 37 °C for 3 h, the absorbance at the wavelength of 450 nm was read by a multifunctional microplate reader (MultiskanMK3; Thermo Scientific, Atlanta, GA). Cell inhibition rate (CIR, %) was calculated by the following formula. The half-inhibitory concentration (IC_50_) values were calculated accordingly.

$${\text{Cell inbibition rate }}(\% ) = \left( {1 - \frac{{{\text{A}}_{\text{s}} - {\text{A}}_{\text{b}} }}{{{\text{A}}_{\text{c}} - {\text{A}}_{\text{b}} }}} \right) \times 100\%$$

where, A_b_, A_c_ and A_s_ refer to the absorbance of the blank wells, the control wells, and the experimental wells, respectively.

The software CalcuSyn (Biosoft, Ferguson, MO, USA), which applies the median-effect equation of Chou and the CI (combination index) equation of Chou and Talalay, was used to analyze the combined effects of GA and PTX on the growth inhibition of 4T1 cells [37]. The combined effects of GA and PTX can be indicated as follows: CI < 1, synergism; CI = 1, additive effect; and CI > 1, antagonism [38].

### Preparation of NLC

NLCs were prepared by emulsification and solvent evaporation method as described previously [9]. To get the NLCs modified with RGE or R9dGR, the formulated materials used to replace the equivalent quantity of lecithin were added to the oil phase [39], and the process was the same as described previously. GA and PTX, Dir, and Cou-6 were into the oil phase when preparing GA/PTX-loaded NLCs, Dir-loaded NLCs or Cou6-loaded NLCs.

### Characterization of NLC

#### Particle size, zeta potential, and morphology

The particle size, polydispersity index (PDI) and zeta potential of GA/PTX-NLC, RGE-GA/PTX-NLC and R9dGR-GA/PTX-NLC were measured by photon correlation spectroscopy using Zetasizer, Nano ZS (Malvern Instruments, Malvern, UK). All measurements were performed in triplicate. Particle morphology of GA/PTX-NLC, RGE-GA/PTX-NLC and R9dGR-GA/PTX-NLC were studied by transmission electron microscopy (TEM, JEOL, Japan).

#### Entrapment efficiency and drug loading

The encapsulation efficacy (EE) of GA and PTX were determined using ultrafiltration method. EE was determined by measuring the concentration of the drug in the dispersion and the total content of the drug with the ultra performance liquid chromatography (UPLC) methods. The calculation formula of EE is as follows:

$${\text{EE }}(\% ) = (1 - {\text{W}}_{\text{free}} /{\text{W}}_{\text{total}} ) \times 100\%$$

In the above formula, W_free_, and W_total_ are the weight of free PTX and GA, and the total weight of PTX and GA in the NLC, respectively.

Drug loading capacity (DL) was the ratio of the encapsulated drug to total lipid. The calculation formula of DL is as follows:

$$DL \, (\% ) = \left( {\left( {PTX \, + \, GA} \right)_{\text{Encapsulated}} /\left( {Lipid} \right)_{\text{Total}} } \right) \times 100\%$$

In the above formula, (PTX + GA) _Encapsulated_, and (Lipid) _Total_ are the total weight of PTX and GA in the NLC, and total weight of solid and liquid lipids.

UPLC analyses were performed on a Waters Acquity UPLC BEH C18 column (2.1 × 50 mm, 1.7 μm) and Waters VanGuard BEH C18 column (2.1 × 5 mm, 1.7 μm). GA and PTX were determined by an UPLC (Waters) equipped with UV-detector. Chromatographic conditions are as follows: The mobile phase comprised of (A) water (containing 0.1% formic acid), (B) acetonitrile. The temperature of the column oven was maintained at 30 °C and the flow rate was 0.2 mL/min. The injected volume was 3 μL and monitored at a wavelength of 360 nm (GA) and 230 nm (PTX). The gradient elution condition is 0–3 min, A 35–20%; 3–5 min, A 20–6%; 5–9 min, A 6–2%. The specificity, linearity, precision and stability of the method have been verified.

### X-ray

PXRD patterns of the different samples were acquired from an X-ray diffractometer (D8 advance, Bruker-axs, Germany). Samples of (a) GA + PTX, (b) Blank-NLC, (c) the physical mixture of NLC’s formulation and GA + PTX, (d) GA/PTX-NLC, (e) RGE-GA/PTX-NLC, (f) R9dGR-GA/PTX-NLC were detected under the following conditions: Cu-Kα tube radiation source, tube pressure 40 kV, current 200 mA, scanning in the 2θ range 5–95°,and scanning rate 5°/min.

### Hemolysis assays and in vitro stability

Hemolysis studies were conducted to assess the safety of NLCs in vivo. Blood was taken from the heart tip of rabbits, and the fibrin was removed by stirring with a glass rod. Then the fibrin removed blood was transferred into a centrifuge tube, and 5 ~ 10 mL normal saline was added. After mixing at 4 °C, the supernatant was centrifuged at 1500 revolutions per minute (RPM) for 10 min, and red blood cells (RBCs) were washed and harvested. Then, the red blood cells were diluted with normal saline into a RBCs suspension with a concentration of 2%. 2% RBCs (w/v) were incubated with the different preparations (a: saline, b: deionized water, c: NLC, d: RGE-NLC, e: R9dGR-NLC, saline solution was used as negative control (0% lysis), and deionized water was used as positive control (100% lysis); the volume ratio of 2% RBCs (w/v) and the preparations is 5:1), shaked well and placed in a constant temperature water bath at 37 °C, and followed by centrifugation at 1500 RPM for 10 min at 1 h, 2 h, 3 h, and 24 h respectively. Then the supernatant was obtained and the absorbance of hemoglobin was measured at 540 nm [40].

$${\text{Hemolysis }}\left( \% \right) = \left[ \left( {{\text{A}}_{ 1} - {\text{A}}_{ 2} } \right)/\left( {{\text{A}}_{ 3} - {\text{A}}_{ 2} } \right) \right] \times 100\%$$

where, A_1_, A_2_ and A_3_ refer to the absorbance of the different preparations, the saline (negative control); and the deionized water (positive control), respectively.

The stability of NLCs in the presence of FBS was determined by a multifunctional microplate reader. Briefly, NLCs were mixed with an equal amount of FBS, then incubated at 37 °C, and oscillated slowly at 30 RPM. According to the predetermined time point (0, 1, 2, 4, 8, 12, 24 and 48 h), 200 μL of the mixture of FBS and NLCs was placed into a 96-well plate, and the transmittance at 750 nm was measured.

### In vitro release profile

In vitro release behavior of Taxol, GA/PTX-Sol, GA/PTX-NLC, RGE-GA/PTX-NLC, and R9dGR-GA/PTX-NLC were performed using the dialysis method. The deionized water containing 0.5% Tween-80 (w/w) at pH 7.4 was selected as the release medium by the reported methods [41]. 5 mL of the preparation was placed into a dialysis bag, which was fastened at both ends, and then the dialysis bag was placed in a vessel containing 250 mL of the releasing medium. The temperature was kept at 37 ± 1 °C, and the rotation speed was set to 100 RPM. 2 mL sample was taken from the release medium at 30 min, 1 h, 2 h, 4 h, 6 h, 8 h, 12 h, 24 h, 36 h, 48 h and then blank release medium of the same temperature and volume was added. The sample was filtered by 0.22 μm filter membrane, and then the contents of GA and PTX of the filtrate were determined by the validated UPLC methods as described in ‘Drug encapsulation efficiency and drug loading capacity’. The release studies were performed in triplicate.

### Cellular uptake study

Cellular uptake studies are an essential aspect of evaluating potential drug delivery systems. The uptake of the different formulations by MDA-MB-231 cells (high level αvβ3 and high level neuropilin-1 expression) [42, 43], 4T1 cells (high level αvβ3 and high level neuropilin-1 expression) [44], and MCF7 cells (low level αvβ3 and high level neuropilin-1 expression) [42, 45] were evaluated by the Operetta High Content Imaging System (PerkinElmer, MA, USA) with a relative humidity of 45% at 25 °C and a 20× objective using Cou-6 as a fluorescent probe at non-toxic concentration. To evaluate the cellular uptake; 4T1 cells, MDA-MB-231 cells, MCF7 cells were seeded in 96-well plate. After 24 h of incubation, the former cell culture medium was replaced with fresh culture medium containing Cou-6-Sol, Cou-6-NLC, RGE-Cou-6-NLC and R9dGR-Cou-6-NLC with an equivalent concentration of Cou-6 for 4 h, 8 h and 12 h. And then 50 μL of mixture containing 3 μg/mL Hoechst 33342 was added to each well to stain the nuclei. Cells were incubated at 37 °C in the dark for 30 min. Next, 100 μL PBS was used to wash three times, and 100 μL of RMPI-1640 medium was added to each well. The fluorescent images were captured in each confocal scan by the Operetta HCA system. The mean fluorescence intensity values of images of each well were calculated using Columbus (PerkinElmer).

### Deep penetrating ability in tumor spheroids

The MDA-MB-231 cells were plated with 200 μL of 1.25 × 10^4^ cells per well onto Corning^®^ 96 Well Spheroid Microplates (Corning Cat. No. 4515), and then the plate was incubated for 72 h at 37 °C in a CO_2_ incubator. The formation of spheroids was monitored using an optical microscope. After 3 days, when the diameter of MDA-MB-231 spheroids reached ~ 500 µm, the 3D-spheroids were incubated for 8 h in culture medium containing Cou-6-Sol, Cou-6-NLC, RGE-Cou-6-NLC and R9dGR-Cou-6-NLC at an equivalent concentration of Cou-6. Subsequently, the spheroids were washed with PBS, fixed with 4% paraformaldehyde for 30 min. The fluorescent images were captured using Confocal Laser Scanning Microscopy by the Z-stacking mode (z 1–8, slice thickness 10 µm) under 20× magnification.

### In-vivo bio-distribution study

In-Vivo Imaging System (IVIS) was used to evaluate the tumor targeting efficiency of the RGE and R9dGR modified NLCs in BALB/c nude mice bearing tumor, and Dir was used as a fluorescent probe. Briefly, 0.1 mL of cell suspension containing 2 × 10^7^ cells/mL the mouse mammary carcinoma 4T1 cells was subcutaneously injected into the right flank of female BALB/c nude mice. The mice were randomly divided into saline group, Dir-Sol group, Dir-NLC group, RGE-Dir-NLC group and R9dGR-Dir-NLC group (6 mice in each group) when the tumor volume grew up to 200 mm^3^ (tumor volume V = ab^2^/2; a: tumor length, b: tumor width). After Dir-Sol and Dir labeled NLCs (0.5 mg/kg Dir) were administered for 4 h, 8 h, 24 h and 36 h, the fluorescence intensity of tumor of each mouse was measured at an excitation wavelength of 710 and emission wavelength of 790 nm using the living body imaging system. In addition, after 24 h of imaging, three mice were sacrificed per group, and the tumor, heart, liver, spleen, lung, and kidney were collected. The fluorescence intensity of tumor and each organ were observed.

### Cytotoxicity study

Measuring the cytotoxicity of GA solution, PTX solution, GA/PTX solution, blank NLC, GA-NLC, PTX-NLC, GA/PTX-NLC, RGE-GA/PTX-NLC, and R9dGR-GA/PTX-NLC against the mouse mammary carcinoma 4T1 cells was performed by CCK-8 assay using the similar methods as described in ‘Determination of combination index’.

### P-gp activity assay

The activity of P-gp was determined using a fluorometric MDR assay kit (Cayman, Ann arbor, USA). The MCF-7 and MCF-7/ADR cells were inoculated on a 96-well culture plate and incubated for 24 h. And then the MCF-7 and MCF-7/ADR cells were exposed with different concentrations of GA for 90 min. The P-gp inhibitor verapamil was used as a positive control. Then 100 μL of the prepared Calcein AM solution was added to each of sample well and incubated for an additional 30 min at 37 °C in dark. Intracellular fluorescence intensity was analyzed by a fluorescence microplate reader (Tecan Safire2, Switzerland) with λex = 485 nm and λem = 535 nm.

The cellular accumulation of rhodamine 123 (Rh-123, known as classic P-gp substrates) was taken as another indicator of P-gp activity in this study [33, 38]. Rh-123 cell uptake was measured in the presence or absence of GA. Verapamil was also positive control. The MCF-7 and MCF-7/ADR cells were inoculated on a 96-well culture plate and incubated for 24 h. The medium was replaced with fresh cell medium containing verapamil or various concentrations of GA for 90 min. Then, the MCF-7 and MCF-7/ADR cells were treated with the solutions of 5.0 μmol/L Rh-123 in RPMI 1640 media for 0.5 h. Then the supernatant containing Rh-123 was removed and the cells are washed twice with cold PBS to remove all the probe traces on the cell surface. Intracellular fluorescence intensity was analyzed by a fluorescence microplate reader with λex = 485 nm and λem = 535 nm.

### Scratch wound‑healing assay

MDA-MB-231 cells were inoculated on 6-well culture plates. 24 h after seeding, the monolayer cells were wounded by 200 μL micropipette tip, washed three times with PBS to remove cell debris, and cultured in cell medium in the absence or presence of GA to 24 h. The wound migration assay of cells treated with GA-NLC, RGE-GA-NLC, and R9dGR-GA-NLC were also carried out. The cell migration rate of the control group was considered as 100%, and that of the other wells was compared with that of the control group. Image J software was used to calculate the scratch area before and after GA incubation.

### Transwell migration and invasion assay

The cell migration and invasion assays were conducted using Transwell plate with 24-well, 8.0 μm pore size Transparent PET Membrance (Corning, USA). For the chamber migration, the upper chambers were inoculated with 0.2 mL MDA-MB-231 cell suspension in serum-free medium and 0.8 mL completed medium were added to the bottom chambers. After the cells attachment, the medium was replaced with fresh cell medium containing proper concentration GA. After 24 h of incubation, 4% paraformaldehyde was used to fix the upper chambers for 25 min. The cotton balls were used to wipe the upper cells of chambers. The lower migrated cells were stained using crystal violet for 30 min for photographed, and the migrated cells were counted as follow: the lower migrated cells stained with crystal violet were treated with ultrasound for 10 min in 35% glacial acetic acid to fully dissolve the crystal violet, and then 200 μL dissolvent was placed in 96-well plates and measured by a multifunctional microplate reader at 570 nm wavelength. The cell migration rate of the control group was considered as 100%, and that of the other wells was compared with that of the control group. The migration assay of cells treated with GA-NLC, RGE-GA-NLC, and R9dGR-GA-NLC were also carried out.

For invasion assays, the assay was performed in the the same type of steps as the chamber migration assays described above except that Matrigel (20 μL Matrigel and 80 μL serum-free RMPI-60 medium) coated PET Membrance was used. After 24 h of incubation with cell medium containing proper concentration GA, the invading cells were stained for photographed. Similarly, the invasion assay of cells treated with GA-NLC, RGE-GA-NLC, and R9dGR-GA-NLC were also carried out.

### In vivo anti-tumor effect

The mice bearing tumor was obtained as described in ‘In-vivo bio-distribution study’. The mice were randomly divided into saline group, Taxol group, GA-Sol group, PTX-Sol group, GA/PTX-Sol group, GA-NLC group, PTX-NLC group, GA/PTX-NLC group, RGE-GA/PTX-NLC group, and R9dGR-GA/PTX-NLC group (6 mice in each group) when the tumor volume grew up to 100 mm^3^. The mice of saline group were injected with 100 μL saline for the control group. For the Taxol group, 100 μL of Taxol (PTX: 1.7 mg/kg) was injected as the positive drug group. And 100 μL/mice (GA: 2 mg/kg, PTX: 1.7 mg/kg) was injected into tail vein, once every 2 days, 7 times, for the other groups. The weights of mice were obtained using an electronic scale and the tumor volume was measured every 3 days using a Vernier caliper. Finally, 24 h after the last administration, blood samples of mice were collected to measure the biochemical markers of heart functions (CK), hepatic functions (AST and ALT), and kidney functions (CRE). Then the mice were sacrificed by cervical dislocation, and the major organs, and the tumor tissue were collected and weighed.

Tumor inhibition ratio (TIR), which was calculated using the follow formula:

$${\text{TIR}}\left( \% \right)\, = \,\left( { 1- {\text{tumor weight of treated group}}/{\text{tumor weight of control group}}} \right)\, \times \, 100\%$$

Tumor growth inhibition (TGI) was also a significant indicator of antitumor efficacy in vivo, calculated using the follow formula:

$${\text{TGI }}(\% ) = \left( {1 - \frac{{{\text{T}} - {\text{T}}_{0} }}{{{\text{C}} - {\text{C}}_{0} }}} \right) \times 100\%$$

In the formula above, T_0_ and T are the average tumor volume before treatment and on the last day of treatment in the treated group, respectively. C_0_ and C were the average tumor volume before and after treatment in the control group.

In addition, the inhibition rate of body weight (IRBW) was calculated using the follow formula:

$${\text{IRBW }}(\% ) = \left( {1 - \frac{{{\text{BW}}_{\text{a}} }}{{{\text{BW}}_{\text{b}} }}} \right)\times 100\%$$

where BW_a_ and BW_b_ are the body weight of the mice after and before the treatment, respectively.

All the samples of the tumor tissue and the major organs added into 10% formalin for fixation, dehydrated, embedded in paraffin, sectioned, stained with hematoxylin and eosin, were observed under a microscope (NIKON Eclipse Ci). Slides of the tumor sections were deparaffinized, rehydrated and incubated with Ki67 antibody, CD31 antibody, TUNEL detection kit (Roche) for immunofluorescence staining. As for TUNEL assay, DAPI was added to stain the nuclei. The samples were observed under an inverted fluorescence microscope (Nikon Eclipse Ti-SR).

### Pharmacokinetic study in mice

In vivo pharmacokinetic studies were conducted in BALB/c female mice. The pharmacokinetic behavior of Taxol^®^, GA/PTX-Sol, GA/PTX-NLC, RGE-GA/PTX-NLC, and R9dGR-GA/PTX-NLC were evaluated as follows. The mice were randomly divided into four group (n = 3 per group) and 100 μL/mice (GA: 8 mg/kg, PTX: 6.8 mg/kg) was injected into tail vein. Subsequently, blood samples of mice were collected from the ocular vein each animal into heparin-containing tubes at 2 min, 30 min, 1 h, 4 h, 8 h, 24 h, 36 h. Plasma sample was treated by centrifugation at 10,000 RPM for 10 min and stored at − 80 °C. Aliquots (100 μL) of the plasma samples were spiked with 800 μL of acetonitrile containing carbamazepine (200 ng/mL, as an internal standard) for deproteinization. After vortex-mixing for 5 min, the mixture was centrifuged at 10,000 RPM for 15 min. The supernatant was then carefully transferred to another tube, evaporated under nitrogen flow. The residue was reconstituted with mobile phase.

UPLC-MS/MS analyses were conducted on a Waters Acquity UPLC BEH C18 column (2.1 × 50 mm, 1.7 μm). The mobile phase comprised of (A) water (containing 2 mM ammonium formate), (B) acetonitrile. The temperature of the column oven was maintained at 40 °C and the flow rate was 0.3 mL/min. Then 5 μL was injected into the UPLC-MS/MS system. The UPLC gradient program was set as follows: 0-4 min, A 80–60%; 4–6 min, A 60–0%; 6–7.5 min, A 0–0%; 7.5–8 min, A 0–80%. The analytes are detected using an electrospray positive ionization (ESI^+^) with the multiple reaction monitoring mode (MRM). The ESI configuration was as follows: gas flow rate 11 l/min; gas temperature 350 °C; nebulizer 20 psi; capillary 4000 V (Agilent Technologies). The mass transition ion-pair was selected as m/z 628.76 → 572.7 for GA, m/z 876.0 → 307.8 for PTX and m/z 237.1 → 193.8 for IS. The specificity, linearity, precision, recovery, matrix effect and stability of the method have been verified. The pharmacokinetic parameters of each formulation, terminal half-life (t_1/2_), mean residence time (MRT), and time-averaged total body clearance (CL), area under the drug concentration in plasma–time curve from time zero to infinity (AUC) were calculated using WinNonlin 8.2.

### Statistical analysis

The all values were represented as mean ± standard deviation (SD). Statistical significance was analyzed using IBM SPSS Statistics 22.0 software by one-way ANOVA or Student’s t-test with the p value < 0.05 or p value < 0.01 or p value < 0.001 indicating significance.

## Results and discussion

### Determination of combination index

**Fig. 1:**
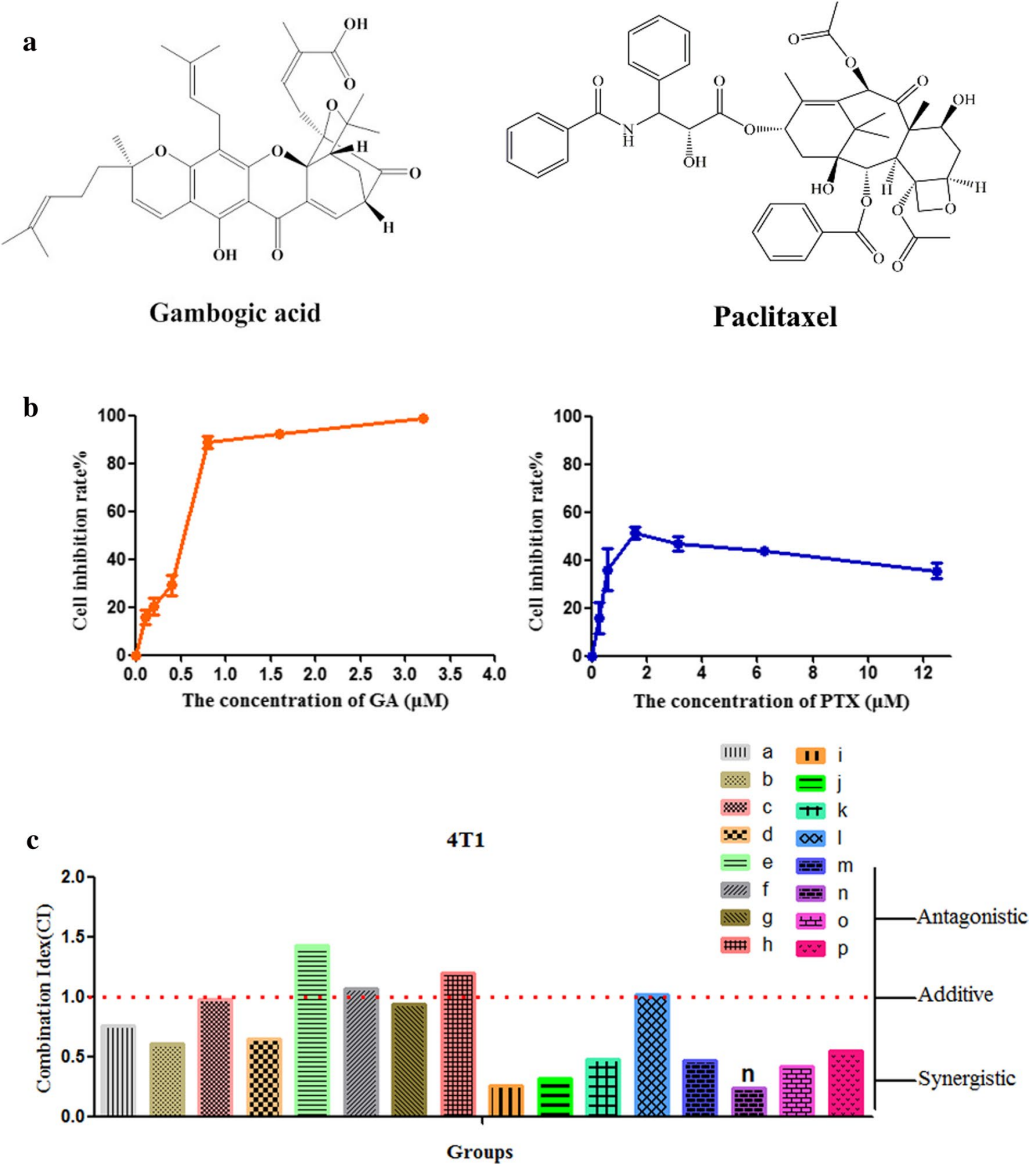
Effect of GA and PTX on the growth of 4T1 cells in vitro. **a** The chemical structure of GA and PTX. **b** GA and PTX dose–response curves for cell viability (mean ± SD, n = 3), μM stands for μmol/L. **c** CI value of GA combined with PTX (*a*–*p* was shown in Table 1)

The anti-proliferative effects of GA and PTX were determined with the CCK-8 assay in 4T1 cell lines after 24 h of drug treatment, and the interaction between GA and PTX was quantified by measuring the CI. As a first step, the cytotoxicities of GA and PTX in 4T1 cells were respectively examined, and then synthetic effects on 4T1 cells were evaluated. As seen in Fig. 1b, GA and PTX have a significant inhibitory effect on the growth of 4T1 cells with 24 h of incubation. The PTX incubation exhibited the cytotoxicity against the mouse mammary carcinoma 4T1 cells with an estimated IC_50_ of 1.19 ± 0.22 μmol/L. The proliferation of 4T1 cells was inhibited by GA in a concentration-dependent manner, with an IC_50_ at 0.67 ± 0.09 μmol/L.

To measure the CI of GA and PTX, we exposed 4T1 cells to a series of concentrations of GA below its IC_50_ (0.065 μmol/L, 0.135 μmol/L, 0.27 μmol/L, 0.4 μmol/L) in combination with lower dose (0.125 μmol/L, 0.25 μmol/L, 0.5 μmol/L, 0.75 μmol/L) of PTX for subsequent studies. The results showed that CI values of different combinations of drug doses ranged from 0.240 to 1.436 as seen in Table 1 and Fig. 1c, which showed that no synergistic cytotoxiciity was observed in e, f, h, l (CI>1). Analysis of the rest of groups (a, b, c, d, g, i, j, k, m, n, o, p; CI<1) indicated synergism, and GA and PTX showed significantly synergistic anti-tumor effects, and a greater cytotoxicity of GA combine with PTX was observed in 4T1 cells compared with treatments using GA or PTX alone. Among them, GA = 0.4 μmol/L, PTX = 0.25 μmol/L had the smallest CI index and CI = 0.240, indicating the strongest synergy among 16 groups, as depicted in the Fig. 1c. This particular combination dose with the lowest CI value (n, GA = 0.4 μmol/L, PTX = 0.25 μmol/L, the molar ratio of GA/PTX was 1.6:1; the weight ratio is GA/PTX = 1.18:1) was chosen for further studies. Hence GA/PTX at 1.18:1 calculated mass ratio was selected to be entrapped into NLCs and be further investigated.

**Table 1:** CI values of GA combined with PTX

	PTX = 0.125 μmol/L	PTX = 0.25 μmol/L	PTX = 0.5 μmol/L	PTX = 0.75 μmol/L
GA = 0.065 μmol/L	0.758 (a)	0.615 (b)	0.982 (c)	0.654 (d)
GA = 0.135 μmol/L	1.436 (e)	1.067 (f)	0.946 (g)	1.200 (h)
GA = 0.27 μmol/L	0.258 (i)	0.325 (j)	0.478 (k)	1.022 (l)
GA = 0.4 μmol/L	0.474 (m)	0.240 (n)	0.424 (o)	0.552 (p)

### Particle size, zeta potential, and morphology

**Fig. 2:**
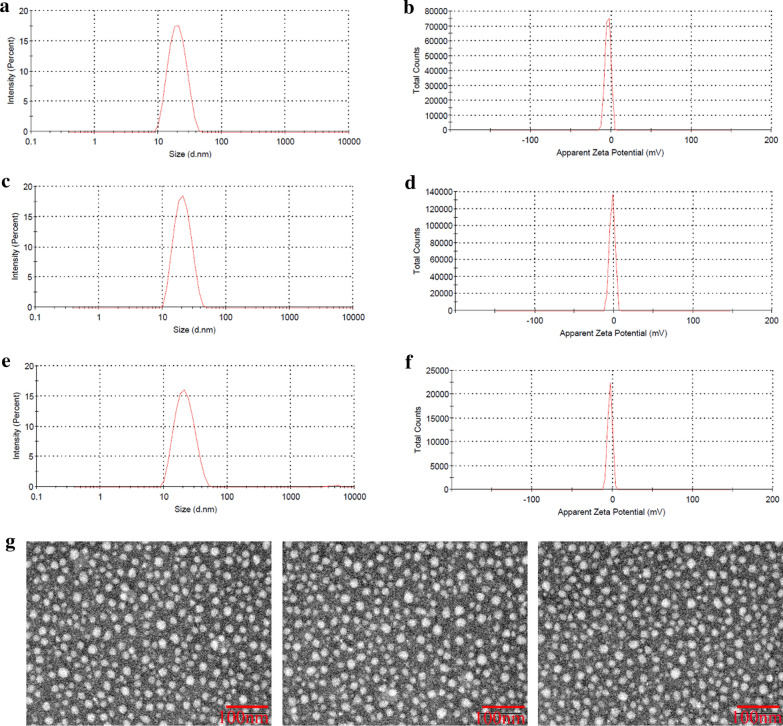
The size and morphology of the formulations. **a**–**f** are particle size distribution and zeta potential of GA/PTX-NLC, RGE-GA/PTX-NLC, and R9dGR-GA/PTX-NLC, as determined by dynamic light scattering. **g** represents the TEM images of GA/PTX-NLC, RGE-GA/PTX-NLC, and R9dGR-GA/PTX-NLC (scale bar = 100 nm)

As shown in Table 2, the particle size of GA/PTX-NLC, RGE-GA/PTX-NLC and R9dGR-GA/PTX-NLC were about 20 to 22 nm with the PDI ranges 0.14 to 0.23, and the zeta potential was between − 2.06 and − 3.87 mV, indicating the size distribution of NLCs is acceptable for the application of targeted therapy of breast cancer [10]. Typical particle size distribution and zeta potential by dynamic light scattering for the prepared GA/PTX-NLC, RGE-GA/PTX-NLC and R9dGR-GA/PTX-NLC were shown in Fig. 2a–f. TEM was used to evaluate the morphological characteristics of the NLCs. In morphology, the NLCs were quasi spherical and well-dispersed (Fig. 2g). In general, the morphological study of GA/PTX NLCs and targeted GA/PTX NLCs revealed that all of the NLCs were spherical in shape with a uniform particle size.

**Table 2:** Particle size, polydispersity index, zeta potential, EE, and DL of GA/PTX-NLC, RGE-GA/PTX-NLC and R9dGR-GA/PTX-NLC (mean ± SD, n = 3)

Preparation	Size (nm)	PDI	ZP (mV)	EE % (GA)	EE % (PTX)	DL % (GA)	DL % (PTX)
GA/PTX-NLC	20.92 ± 0.56	0.14 ± 0.04	− 3.87 ± 1.50	98.69 ± 0.46	97.88 ± 0.32	7.46 ± 0.03	6.37 ± 0.04
RGE-GA/PTX-NLC	21.58 ± 0.63	0.19 ± 0.09	− 2.06 ± 0.69	97.54 ± 0.71	98.18 ± 0.12	7.39 ± 0.04	6.31 ± 0.03
R9dGR-GA/PTXNLC	22.03 ± 0.48	0.23 ± 0.06	− 2.88 ± 0.96	98.17 ± 0.43	97.69 ± 0.21	7.55 ± 0.06	6.27 ± 0.04

### Entrapment efficiency and drug loading

The EE of NLCs for GA/PTX were investigated when the molar ratio of GA/PTX was fixed as 1.6:1 (weight ratio was 1.18:1), and were determined by UPLC method. The experimental result showed that GA and PTX were simultaneously encapsulated in the CPP modified GA/PTX-NLC or unmodified GA/PTX-NLC with EE of >97%, which significantly improved the solubility of GA and PTX. No significant difference was observed for the DL and EE in the untargeted and targeted NLCs, indicating that CPP surface modification has no effect on DL and EE of GA/PTX-NLC.

### X-ray

**Fig. 3:**
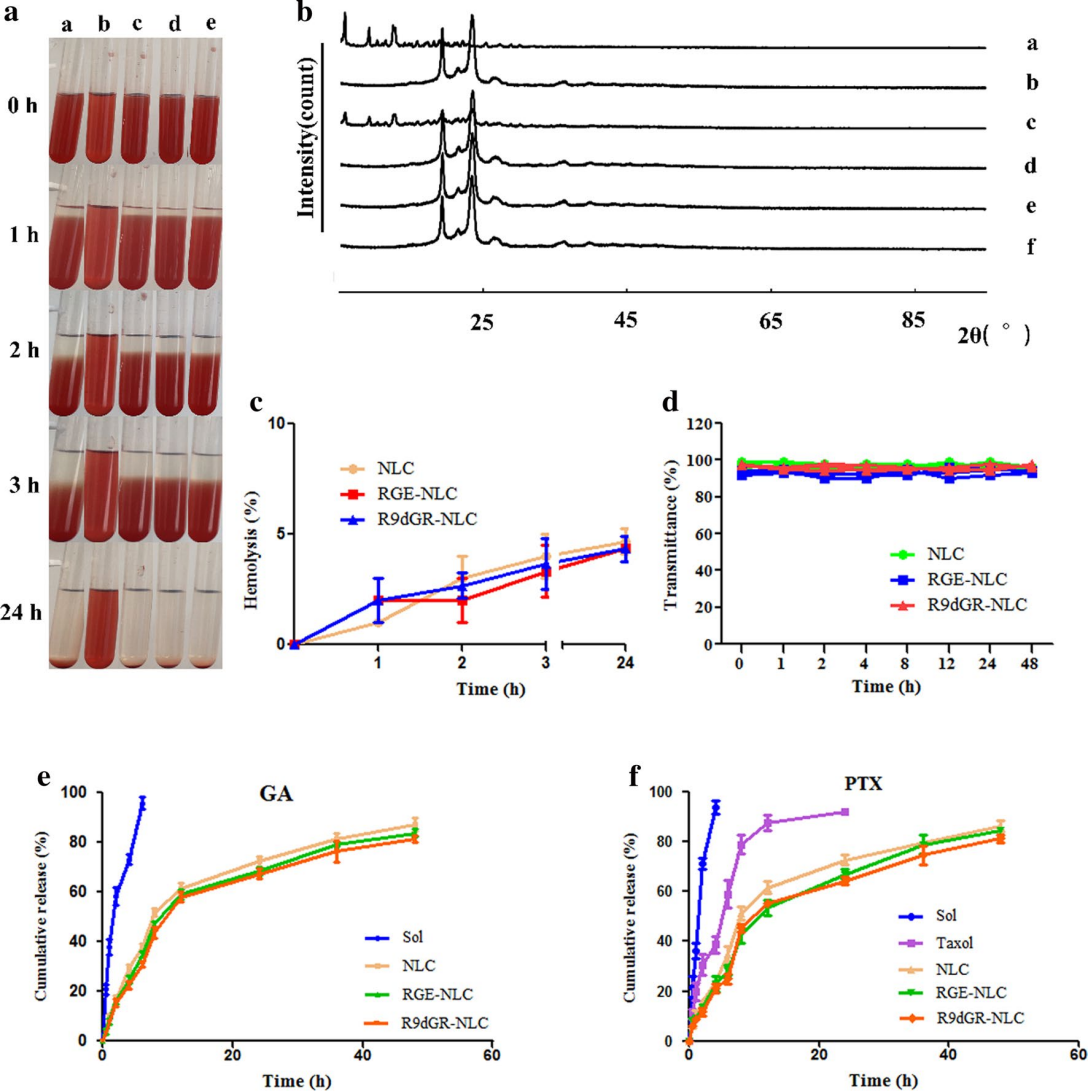
**a** Hemolysis assays of NLCs (*a*: saline, *b*: deionized water, *c*: NLC, *d*: RGE-NLC, *e*: R9dGR-NLC). **b** X-ray of different formations (*a*: GA + PTX, *b*: Blank-NLC, *c*: the physical mixture of NLC’s formulation and GA + PTX, *d*: GA/PTX-NLC, *e*: RGE-GA/PTX-NLC, *f*: R9dGR-GA/PTX-NLC). **c** The quantitative results of hemolysis assay of NLCs in vitro (mean ± SD, n = 3). **d** The variations of transmittance of NLCs in 50% FBS over 48 h (mean ± SD, n = 3). **e**, **f** GA and PTX release profiles from different formulations in vitro (mean ± SD, n = 3)

PXRD was used to obtain more information on crystal structural properties. Figure 3b shows the comparative PXRD patterns: GA and PTX (a); Blank-NLC(b); GA, PTX, and the physical mixture of NLC’s formulation (c); GA/PTX-NLC (d); RGE-GA/PTX-NLC (e); and R9dGR-GA/PTX-NLC (f). As can be seen from the Fig. 3b, there were obvious characteristic peaks of the standard mixture of GA + PTX at a diffraction angle (2θ) of 5.633°, 9.282°, 10.680°, 12.366°, and 13.480°. The freeze-dried Blank nanostructured lipid carrier (Blank NLC) had obvious characteristic peaks at a diffraction angle (2θ) of 19.180°, 21.840°, 23.280° and 26.340°. These characteristic peaks were observed in the freeze-dried samples of the physical mixtures (blank lipid carrier samples, GA and PTX), indicating that GA/PTX existed as a crystalline state in the physical mixture. In GA/PTX-NLC, RGE-GA/PTX-NLC and R9dGR-GA/PTX-NLC samples after freeze-drying, the characteristic peaks of GA and PTX disappeared, which confirmed that GA and PTX were encapsulated in the nanostructured lipid carrier and there was no crystallisation of drug inside the lipid nanocarrier matrix, indicating the change of GA/PTX from crystalline nature to an amorphous state [46].

### In vitro stability and hemolysis assays

No aggregation was observed in the untargeted and targeted NLCs after incubation with FBS for 48 h (Fig. 3d), which demonstrated the satisfactory serum stability of GA/PTX-NLC, RGE-GA/PTX-NLC, and R9dGR-GA/PTX-NLC. The biocompatibility of nanoparticles could be evaluated by the hemolysis assay [47]. The results showed that a, c, d, and e groups showed no hemolysis or agglutination, and evenly dispersed after shaking, while b group showed complete hemolysis for the rupture of red blood cells (Fig. 3a). The quantitative results showed that there was no hemolysis and agglutination of rabbit RBCs on the GA/PTX-NLC, RGE-GA/PTX-NLC, and R9dGR-GA/PTX-NLC, which could be used for injection (Fig. 3c).

### In vitro release profile

The release of GA and PTX from the untargeted and targeted NLCs showed a similar pattern, and there was no significant difference in the release of all groups of NLCs in vitro. The similar physicochemical properties of targeted and non targeted NLCs provided the basis for later research [47]. However, compared with GA/PTX-Sol, and Taxol, the release time of NLCs groups were significantly prolonged (Fig. 3e and f). The sustained release of GA and PTX from different NLCs is mainly due to the gradual degradation of nanoparticles.

### Cellular uptake study

**Fig. 4:**
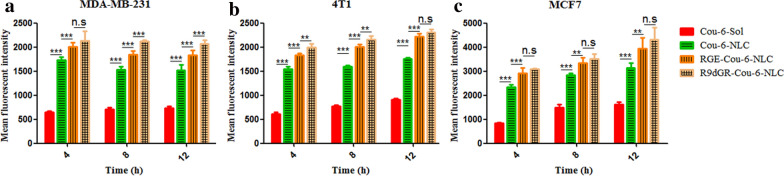
The fluorescence intensity of Cou-6-Sol, Cou-6 labeled NLC and CPP modified Cou-6 labeled NLC (Cou-6 at the concentration of 0.05 μg/mL) at different incubation time interval (4 h, 8 h, and 12 h) (**a**: MDA-MB-231 cells, **b**: 4T1 cells, **c**: MCF-7 cells). *P < 0.05; **P < 0.01; ***P < 0.001; n.s., not significant. Results are expressed as mean ± SD, n = 3.

Cou-6, a green fluorescent marker with a high fluorescent intensity and a low leakage rate [48], was selected for cell uptake study with an appropriate concentration (0.05 μg/mL) as described previously [10]. The cytotoxicity of Cou-6 labeled NLC for cell uptake was determined, which has no effect on cell activity (data no shown). The cell uptake of Cou-6-Sol and various Cou-6-loaded NLCs at equivalent Cou-6 concentrations in 4T1, MDA-MB-231, and MCF7 cells was shown in Fig. 4. In 4T1, MDA-MB-231, and MCF7 cells, at all-time points, the intake of polypeptide modified nanoparticles exhibited significantly higher uptake than that of solution and unmodified nanoparticles, namely the fluorescent intensity: CPP-Cou-6-NLC > Cou-6-NLC > Cou-6-Sol, suggesting the lower internalization efficiency of Cou-6-Sol than Cou-6-NLCs and the modification of CPP elevated the internalization of NLCs significantly. This may be explained by the following reasons: different from the cellular uptake of the Cou-6-Sol by a passive diffusion mechanism, Cou-6-NLCs and CPP-Cou-6-NLC entered the cells through nonspecific endocytosis and receptor-mediated endocytosis, respectively.

The cellular uptake was also evaluated between RGE-Cou6-NLC and R9dGR-Cou-6-NLC. For MDA-MB-231 cells, the cell uptake of R9dGR modified NLCs were greater than that of RGE decorated NLCs after 8 h and 12 h incubation, namely R9dGR-Cou-6-NLC > RGE-Cou6-NLC (P < 0.001) (Fig. 4a). At 4 h, there was no significant difference in intake, possibly due to the shorter incubation time. In 4T1 cells, the same thing happened with respect to cellular uptake after 4 h and 8 h incubation (P < 0.01) (Fig. 4b), and there was no difference between RGE-Cou6-NLC group and R9dGR-Cou-6-NLC group at 12 h, probably owing to receptor-mediated endocytosis reaching saturation. And as for MCF7 cells (Fig. 4c), the cell uptake difference between R9dGR-Cou-6-NLC and RGE-Cou6-NLC were not observed, which may be caused by that RGERPPR has a high affinity toward NRP-1 receptor, and R9dGR could bind to both integrin αvβ3 and NRP-1 receptors, while MCF7 cells have low level integrin αvβ3 expression and high level NRP-1 receptor expression based on confirmed reports [42, 45]. The experimental results showed that the difference of cell uptake in R9dGR-Cou-6-NLC group and RGE-Cou6-NLC group completely corresponded to the disparity of expression level of receptor on different cells. Microscopy images of cell uptake in MDA-MB-231, 4T1, and MCF-7 cells after treatment with Cou-6-Sol, Cou-6-loaded targeted and nontargeted NLC were shown in Additional file 1: Fig. S2.

As expected, R9dGR-Cou-6-NLC demonstrated more efficient intracellular delivery in comparison to RGE-Cou-6-NLC. The cancer cell recognition ability of R9dGR modified NLC was significantly enhanced, and the internalization of R9dGR-Cou-6-NLC was further elevated, which strongly supported our hypothesis that dual receptor recognizing CPP exhibited a crucial role in enhancing the recognition and uptake of cancer cells, producing well therapeutic effect [49].

### Penetration into tumor spheroids

**Fig. 5:**
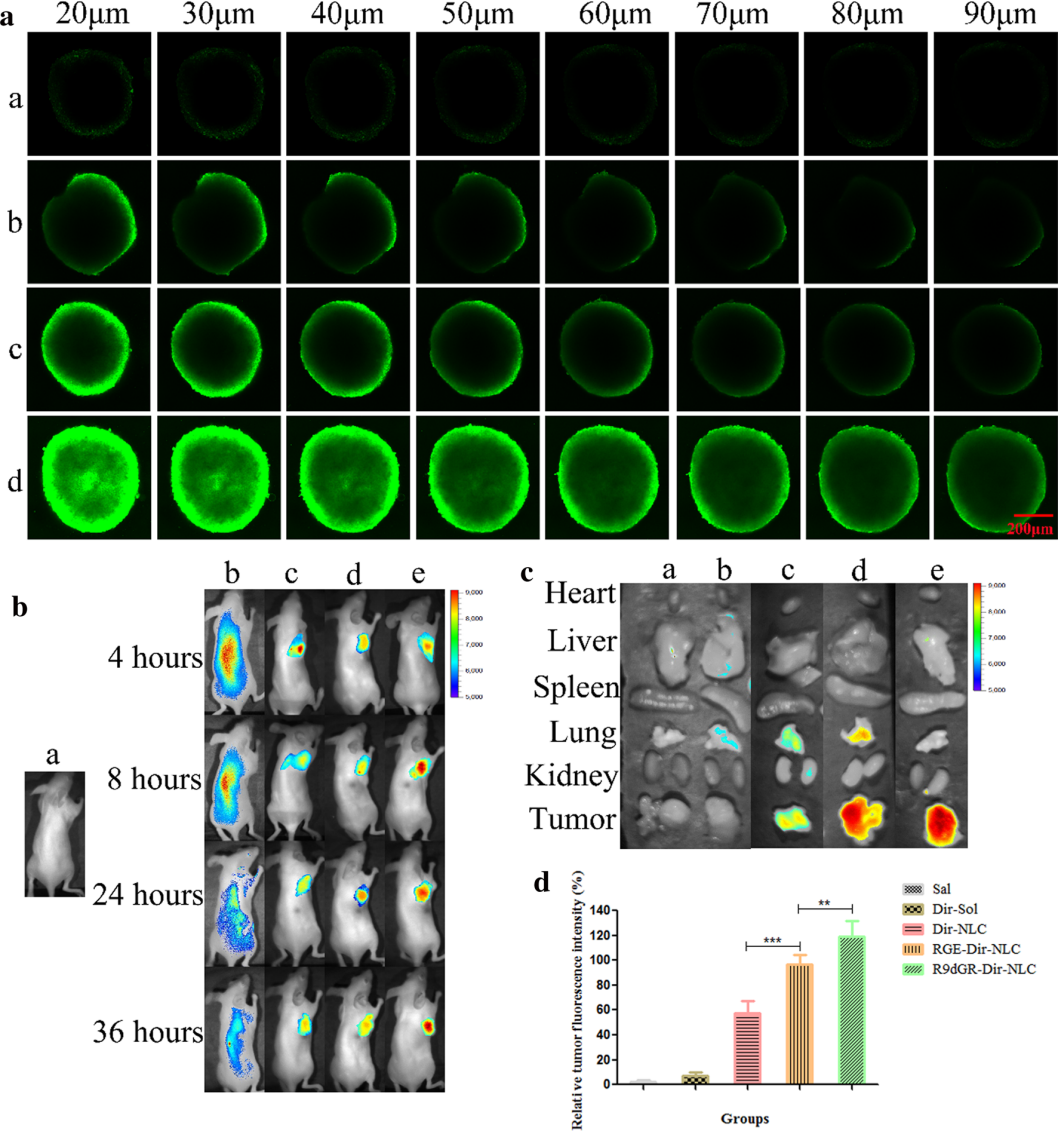
**a** The penetrating ability of Cou-6-labeled NLCs into MDA-MB-231 tumor spheres (*a*: Cou-6-Sol, *b*: Cou-6-NLC, *c*: RGE-Cou-6-NLC, and *d*: R9dGR-Cou-6-NLC), scale bar = 200 μm. **b** The bio-distribution study of Dir-NLC in tumor bearing mice (*a*: Sal, *b*: Dir-Sol, *c*: Dir-NLC, *d*: RGE-Dir-NLC, and *e*: R9dGR-Dir-NLC). **c** Ex-vivo fluorescence images of the organs and tumor tissues excised from mice treated with different Dir formulations (*a*: Sal, *b*: Dir-Sol, *c*: Dir-NLC, *d*: RGE-Dir-NLC, and *e*: R9dGR-Dir-NLC). **d** The statistical results of fluorescence signal of tumor obtained from mice treated with different Dir formulations. *P < 0.05; **P < 0.01; ***P < 0.001. Results are expressed as mean ± SD

As mentioned above the cellular uptake study (two-dimensional (2D) monolayer culture model) in vitro has been widely used, and the related internalization studies provide a prediction for the performance of nano-carrier system in vivo [50]. However, two-dimensional model can not accurately reflect the complexity of real tumor tissue, so the three-dimensional (3D) tumor sphere model is an intermediate model introduced in recent years, which exhibits physiologically relevant cell–cell and cell–matrix interactions, signaling pathways profiles and gene expression, structural complexity and heterogeneity that reflect in vivo tumors [51]. In this study, a 3D tumor spheroid model of MDA-MB-231 cells was prepared as an in vitro evaluation system for the penetrating ability of R9dGR-NLC. As shown in the Fig. 5a, Cou-6-Sol could not penetrate deep into the core area of the MDA-MB-231 spheroid within 8 h, and exhibit a faint fluorescence on the whole sphere. Cou-6-NLC distributed on the surface of the spheroid, exhibiting limited penetration in MDA-MB-231 spheroids model. The exposure of the polypeptide enhanced the penetration of NLC into the tumor spheroid, and R9dGR-Cou-6-NLC displayed the strongest penetration inside the spheroids (the highest Cou-6 accumulation in the core region), followed by RGE-Cou-6-NLC. Taken the cellular uptake results together, the R9dGR-Cou-6-NLC is superior to RGE-Cou-6-NLC and Cou-6-NLC in promoting the cellular uptake and deep penetration in vitro models.

### In-vivo bio-distribution study

As shown in Fig. 5b, it can be seen that there was no significant fluorescence effect in the saline group. Dir-Sol group showed non-specific distribution and rapid clearance in vivo after intravenous administration, and no significant Dir accumulation of Dir-Sol group in tumor sites was observed (Fig. 5b). Dir-NLC, RGE-Dir-NLC and R9dGR-Dir-NLC groups could be clearly observed to have strong DiR fluorescence in tumor tissues after injection 4 h. At 8–24 h, the fluorescence intensity of tumors in each NLCs’ group gradually increased with time. The R9dGR-Dir-NLC group exhibited the strongest fluorescence intensity, followed by RGE-Dir-NLC and Dir-NLC, namely fluorescence intensity: R9dGR-Dir-NLC > RGE-Dir-NLC > Dir-NLC. The accumulation of NLC in tumor sites might be related to EPR effect, while R9dGR-Dir-NLC and RGE-Dir-NLC not only had EPR effect, also had the ability of active targeting, which facilitated the delivery of drugs into the tumor. In order to clearly observe the intensity of Dir fluorescence signal, 24 h after injection, the main organs and tumors were excised and collected. The in vitro imaging results of tumors and organs were shown in Fig. 5c.

It can be seen from the Fig. 5c that the fluorescence signal of Dir was not observed in the tumors of the Dir-Sol group. But the strong Dir fluorescence signal can be observed in the tumor of NLC group. The tumor tissues still have strong Dir fluorescence, indicating that the nanoparticles have obvious tumor targeting. The fluorescence intensity of R9dGR-Dir-NLC was stronger than that of the other groups, indicating that R9dGR-Dir-NLC had strong tumor targeting. The quantitative results were shown in the Fig. 5d. The fluorescence signal of R9dGR-Dir-NLC in tumor tissue is 1.22 times higher than that of RGE-Dir-NLC (P < 0.01), indicating that R9dGR in vivo had stronger targeting ability own to the high affinity of it to both integrin αvβ3 and NRP-1 receptors that were high-expressed on 4T1 cells [44] and mediated the internalization of the nanocarrier into the cell. This finding was consistent with the results of in vitro cell uptake experiments. The heart, liver, kidney and spleen showed no obvious fluorescence signal. However, the accumulation of Dir in the lungs of mice was observed in RGE-Dir-NLC and Dir-NLC groups.

### Cytotoxicity study

The previous experimental results shown that as GA = 0.4 μmol/L and PTX = 0.25 μmol/L, the CI value was the smallest, showing the strongest synergistic anti-tumor effect. Therefore, in this part, the in vitro antitumor activity of PTX-Sol (a), GA-Sol (b), PTX + GA-Sol (c), PTX-NLC (d), GA-NLC (e), GA/PTX-NLC (f), RGE-GA/PTX-NLC (g) and R9dGR-GA/PTX-NLC (h) were determined on 4T1 cells using the concentration above after 24 h incubation.

**Fig. 6:**
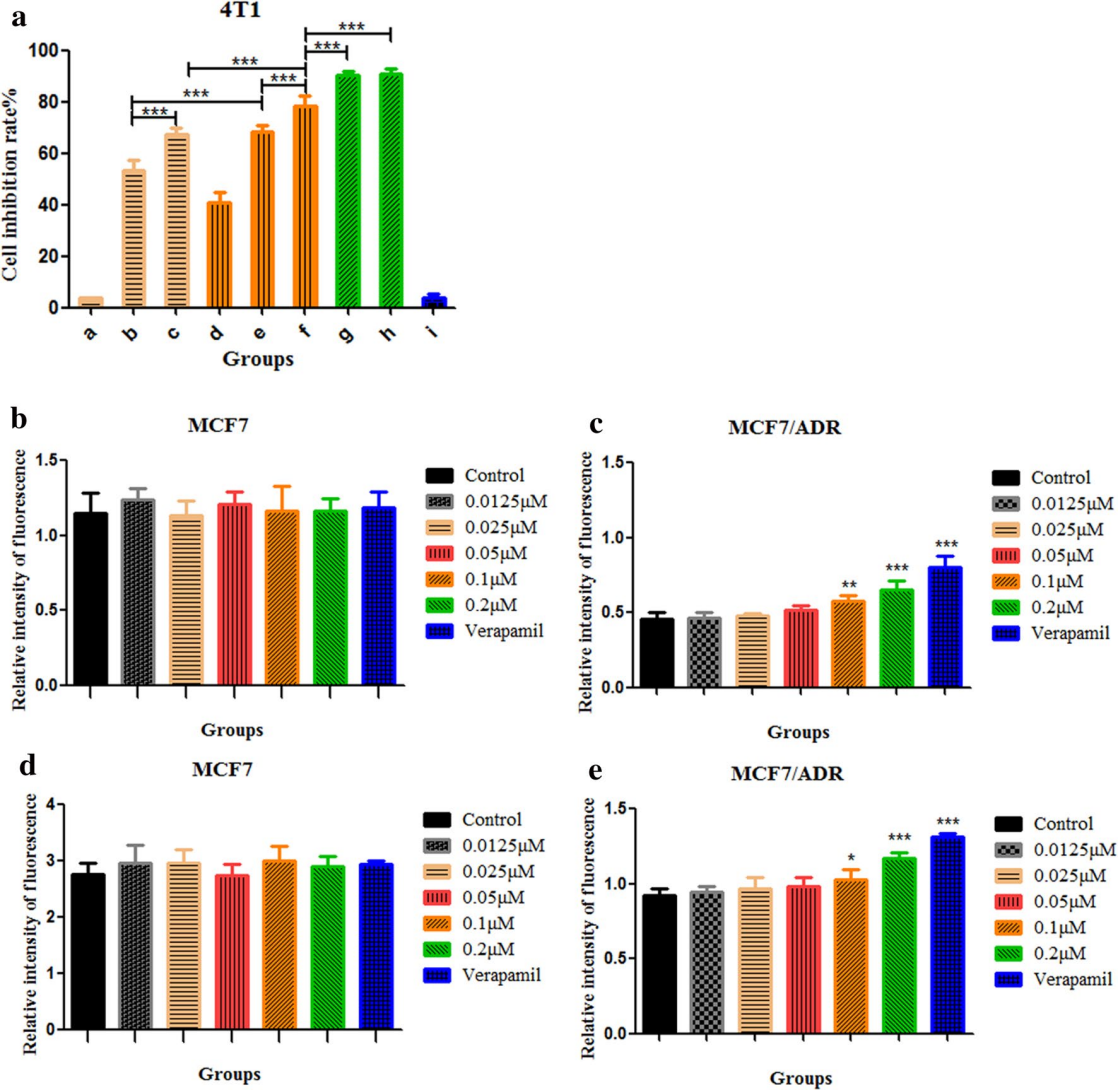
**a** The cell viability of different formulations was analyzed by CCK-8 (*a*: PTX-Sol, *b*: GA-Sol, *c*: PTX + GA-Sol, *d*: PTX-NLC, *e*: GA-NLC, *f*: GA/PTX-NLC, *g*: RGE-GA/PTX-NLC, *h*: R9dGR-GA/PTX-NLC and *i*: blank NLC; GA = 0.4 μmol/L and PTX = 0.25 μmol/L). **b**, **c** MCF-7 and MCF-7/ADR cells was treated with different concentrations of GA and verapamil, then the P-gp activities were determined using a fluorometric MDR assay kit, μM stands for μmol/L. **d**, **e** Effects of GA-Sol and verapamil on cellular accumulation of Rh-123 in MCF-7 and MCF-7/ADR cells, μM stands for μmol/L. Results are expressed as mean ± SD, n = 3

As shown in Fig. 6a, the combination of GA and PTX showed higher cytotoxicity against 4T1 cells compared with the single drug, which was in agreement with the previous results. Meanwhile, the cytotoxicity of GA and PTX encapsulated with NLC increased significantly, indicating that the nanocarrier enhanced the cytotoxicity of GA and PTX, which could be due to the enhanced endocytosis by the tumor cells and it was consistent with the cellular uptake results. Moreover, NLC has the ability to improve the stability of GA and PTX, enhancing the therapeutic efficacy.

The cytotoxicity of the polypeptide modified nanoparticles and unmodified nanoparticles were also evaluated. NLC modified with CPP, the cytotoxicity of g and h was significantly higher than that of the unmodified group f, suggesting that conjugating RGE and R9dGR on the surface of NLC enhanced the uptake of 4T1 cells, thereby increasing the intracellular concentrations of GA and PTX, which ultimately leads to increased cytotoxicity and would be helpful to improve the effect of tumor treatment. However, no significant difference was observed between RGE-GA/PTX-NLC and R9dGR-GA/PTX-NLC, which both has reached about 90% of the cell inhibition rate. In addition, the toxicity of blank NLC (i) to 4T1 cells was negligible, which confirmed the safety of nano-drug carriers. Generally, the cytotoxicity studies demonstrated that RGE and R9dGR modified GA/PTX-NLC have an ideal inhibitory effect on 4T1 breast cancer cells, exhibiting superior in vitro anticancer activity.

### P-gp activity assay

As a distinctive member of the multidrug resistance efflux transporter family, P-gp could reduce the concentration of the drug in the cell by unilaterally transporting intracellular drugs out of cells [52, 53]. As shown in the Fig. 6c, compared with the untreated control group, the intensity of fluorescence in MCF-7/ADR cells exposed with 0.0125, 0.025, 0.05, 0.1, 0.2 μmol/L GA and verapamil (50 μg/mL) for 90 min increased 1.02-, 1.05-, 1.13-, 1.26-, 1.42-, and 1.77-fold respectively, suggesting that the efflux ability of P-gp was inhibited by GA. After GA and verapamil treatment, there were no significant changes in the expression of P-gp in MCF7 (Fig. 6b). The P-gp inhibition assay in vitro was also conducted using Rh-123 as P-gp substrate in MCF-7 and MCF-7/ADR cells to further confirm that GA can block P-gp activity [33]. As shown in Fig. 6e, compared with Rh-123 groups, GA (0.1 and 0.2 μmol/L) + Rh-123 and verapamil + Rh-123 groups exhibited the significantly stronger intensity of fluorescence in MCF-7/ADR. No significant changes of fluorescence intensity in different groups were observed in MCF7 (Fig. 6d). These results suggested that GA could effectively reverse MDR by inhibiting the activity of P-gp, and ultimately lead to the increase of drug accumulation in cancer cells, and improve the therapeutic effect significantly. This finding was consistent with the reported in literature [54].

### Scratch wound-healing assay

**Fig. 7:**
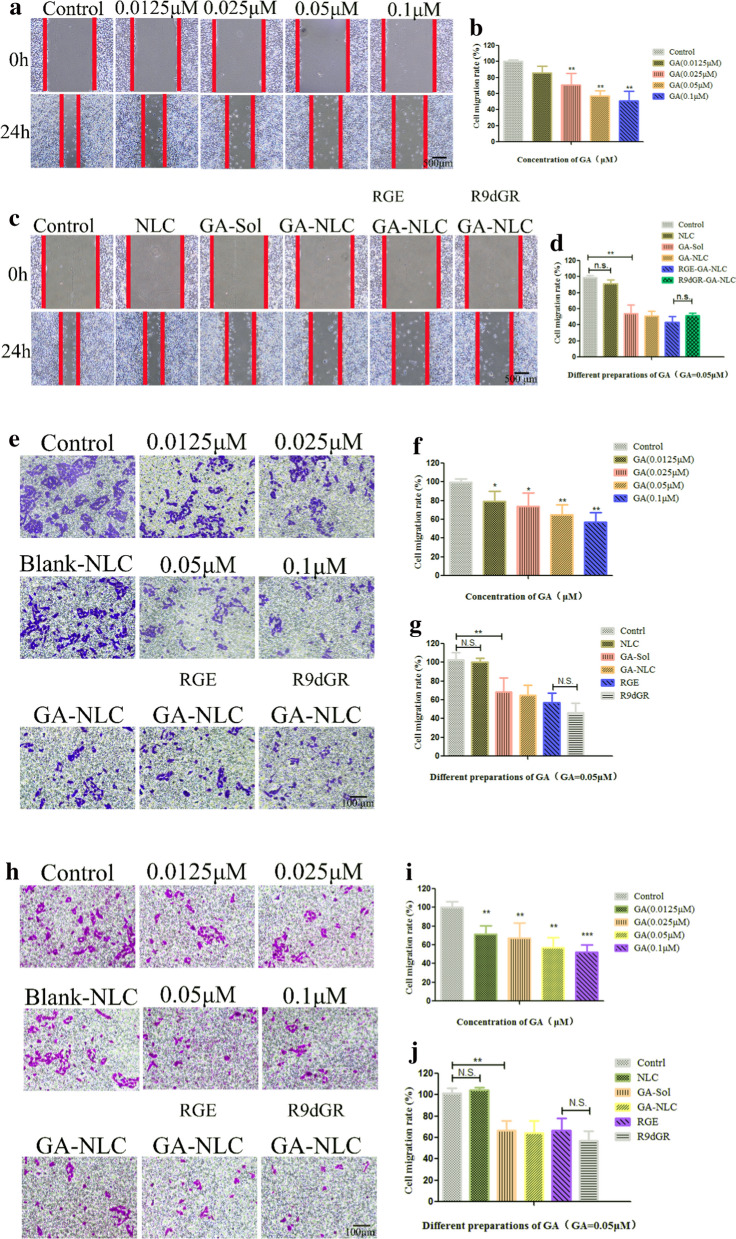
In vitro inhibitory effects of GA-Sol and GA-NLC on cell migration. Typical images of scratch wound-healing assays (**a**, **c**), Transwell migration assay (**e**), and Transwell invasion assay (**h**) performed in MDA-MB-231 cells. The statistical results of scratch wound-healing assays (**b**, **d**), Transwell migration (**f**, **g**) and invasion (**i**, **j**) assays. Scale bar = 500 μm (**a**, **c**), and scale bar = 100 μm (**e**, **h**). The inhibitory effect was normalized to control values, *P < 0.05; **P < 0.01; ***P < 0.001. Results are expressed as mean ± SD, n = 3; μM stands for μmol/L

The cell motility is an indicator of cancer cell metastasis potential. Therefore, we further investigated the effects of GA on cell motility. The cytotoxicity of different concentrations of GA (0.0125, 0.025, 0.05, and 0.1 μmol/L) for scratch wound‑healing assay was determined, which has no effect on cell activity (data no shown). The experimental results showed that GA significantly delayed the process of cells moving into the wound area with concentration of 0.025 μmol/L (P < 0.01). And the higher the concentration, the stronger the inhibitory ability. As the Fig. 7a shown, the control group cells continually migrated into the wound area by 24 h, resulting in extremely narrow scratch width and almost complete fusion of cells on both sides, while the migration of cells in the experimental group was limited in varying degrees. Quantification of the inhibit cell migration showed GA significantly suppressed MDA-MB-231 cell migration at high concentrations (56.9% and 51.0% of cell migrate rate at 0.05 and 0.1 μmol/L, respectively; Fig. 7b). And then, GA with concentration of 0.05 μmol/L was used for subsequent experiments, and the results showed that GA-NLC, RGE-GA-NLC, R9dGR-GA-NLC also showed strong ability to inhibit cell migration. And no significant difference was observed between RGE-GA-NLC and R9dGR-GA-NLC in inhibiting cell migration (Fig. 7c and d).

### Transwell migration assay and invasion assay

The chamber migration assay was also used to confirm the inhibitory effect of GA on MDA-MB-231 cell migration. Figure 7e showed that MDA-MB-231 cells exposed with a series of concentrations of GA (0.0125, 0.025, 0.05, and 0.1 μmol/L) resulted in a significant decrease compared to control cells in cell migration rate to 79.3%, 74.0%, 65.1%, and 57.2% (Fig. 7f), respectively, which indicated that the migration of MDA-MB-231 cells was inhibited by GA in a concentration-dependent manner. Similarly, GA-NLC, RGE-GA-NLC, R9dGR-GA-NLC (GA 0.05 μmol/L) also showed strong ability to inhibit cell migration at the chamber migration assay (Fig. 7g).

The invasion of the tumor cell is a crucial step in the process of cancer metastasis. The study that GA inhibited the invasive activity of MDA-MB-231 cells was conducted using insert chambers with Matrigel coating. Firstly, the effect of GA on the invasion of cells was measured, and as shown in Fig. 7h, GA could suppress MDA-MB-231 invasion with a dose-dependent manner and significantly reduce the number of invasive cancer cells. Quantitative analysis of the invasive activity demonstrated that GA could significantly inhibit the invasion of MDA-MB-231 cells (Fig. 7i). The number of cells passing through Matrigel-coated filters of different groups treated with GA (0.0125, 0.025, 0.05, and 0.1 μmol/L) was reduced to 71.4%, 67.6%, 57.0% and 52.1%, respectively, in comparison with the control group. Similarly, GA-NLC, RGE-GA-NLC, R9dGR-GA-NLC (GA 0.05 μmol/L) also showed strong ability to inhibit cell invasion in Fig. 7j. This result indicated that GA and GA-NLC could suppress the migration and invasion of MDA-MB-231 cells, which indicated GA could serve as a promising drug for the therapy of cancer metastasis [34].

### In vivo anti-tumor effect

**Fig. 8:**
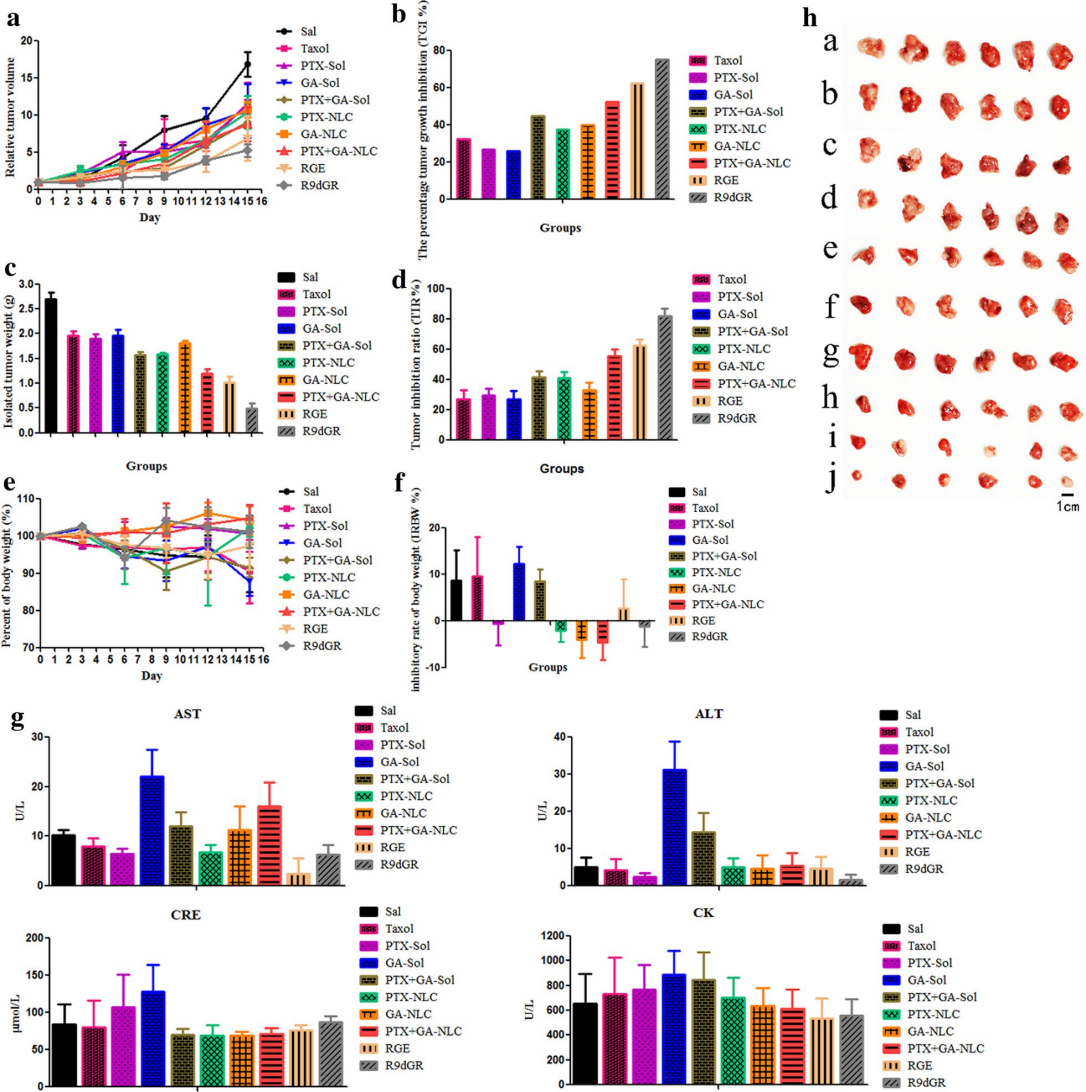
In vivo anti-tumor study of GA and PTX in mice (GA: 2 mg/kg, PTX: 1.7 mg/kg) (**h**; *a*: saline, *b*: Taxol, *c*: GA-Sol, *d*: PTX-Sol, *e*: GA/PTX-Sol, *f*: GA-NLC, *g*: PTX-NLC, *h*: GA/PTX-NLC, *i*: RGE-GA/PTX-NLC, and *j*: R9dGR-GA/PTX-NLC). Results are expressed as mean ± SD, n = 6

Due to the high targeting efficiency of R9dGR-GA/PTX-NLCs, it was expected to obtain better therapeutic effect and lower adverse side effects. The experimental results are shown in Fig. 8a, rapid tumor growth was observed in control group (saline group), however the other treatment groups successfully suppressed the growth of tumor in varying degrees. The tumor volume of GA combined with PTX group was significantly suppressed in comparison with the single drug (P < 0.05). Meanwhile, the tumors grew slowly in the NLC groups compare to solution groups, suggesting the antitumor effect of GA and PTX is enhanced by the nano-carriers. The TGI by Taxol, GA-Sol, PTX-Sol, GA/PTX-Sol, GA-NLC, PTX-NLC, GA/PTX-NLC, RGE-GA/PTX-NLC, and R9dGR-GA/PTX-NLC were 32.07%, 26.84%, 26.02%, 44.52%, 37.34%, 39.80%, 52.13%, 62.31%, and 75.17%, respectively (Fig. 8b). The targeted NLCs were more effective in inhibiting the growth of tumor and exhibited higher antitumor activity, as compared with Taxol, solution groups, and nontargeted NLCs.

As displayed in Fig. 8c and h, the tumor of relatively smaller weight and size was obtained from the mice treated with RGE and R9dGR functionalized GA/PTX-NLC as compared to that of other formulations, and the TIR by Taxol, GA-Sol, PTX-Sol, GA/PTX-Sol, GA-NLC, PTX-NLC, GA/PTX-NLC, RGE-GA/PTX-NLC, and R9dGR-GA/PTX-NLC were 26.92%, 29.57%, 26.81%, 41.50%, 40.99%, 32.66%, 55.39%, 62.32%, and 81.84%, respectively (Fig. 8d). R9dGR-GA/PTX-NLC demonstrated a stronger antitumor efficacy than RGE-GA/PTX-NLC. Among these results, the synergistic antitumor effect of GA and PTX was also well verified. In addition, NLC can increase the tumor suppressive rate of GA and PTX, which is consistent with the above results.

**Fig. 9:**
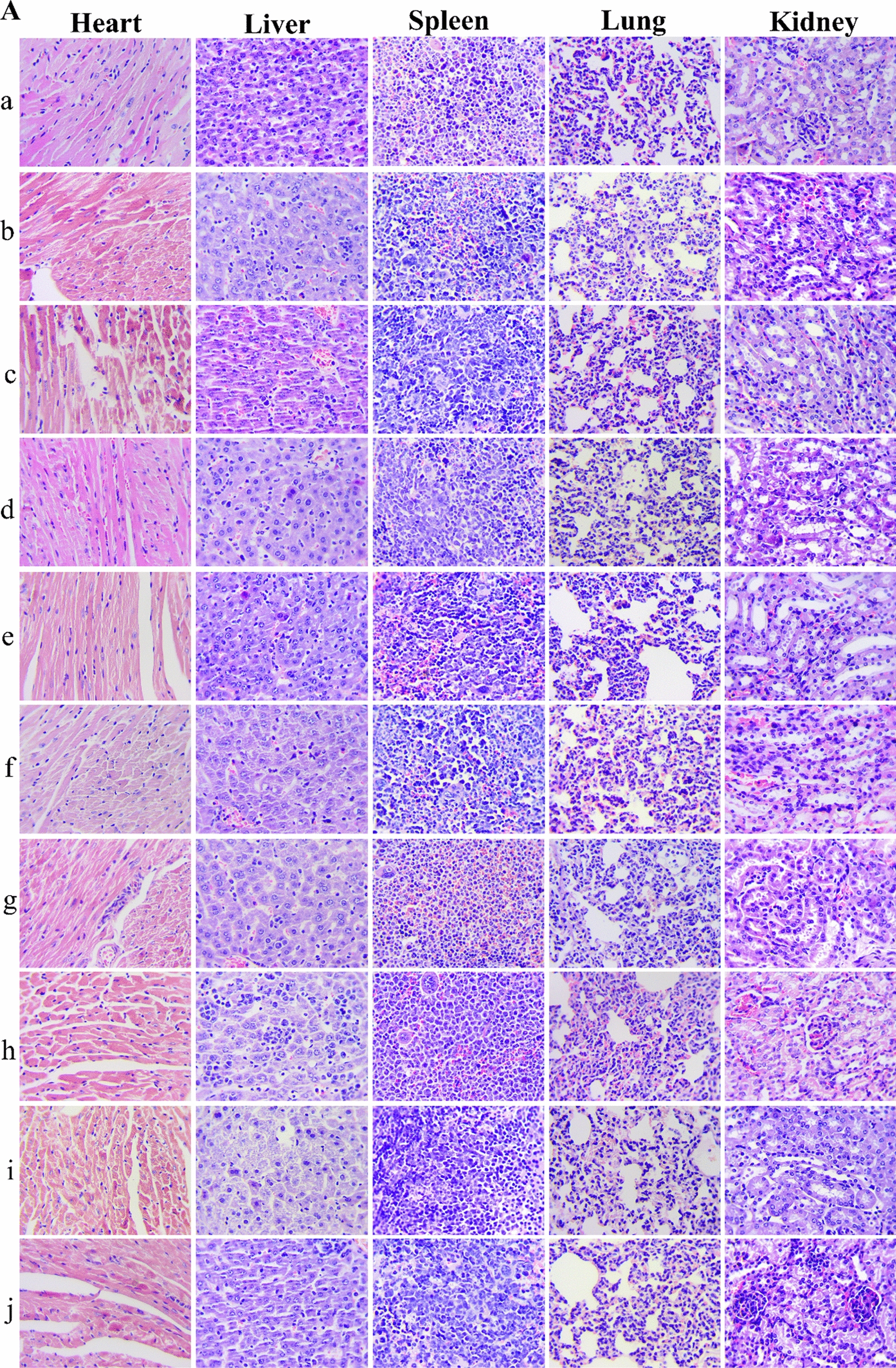

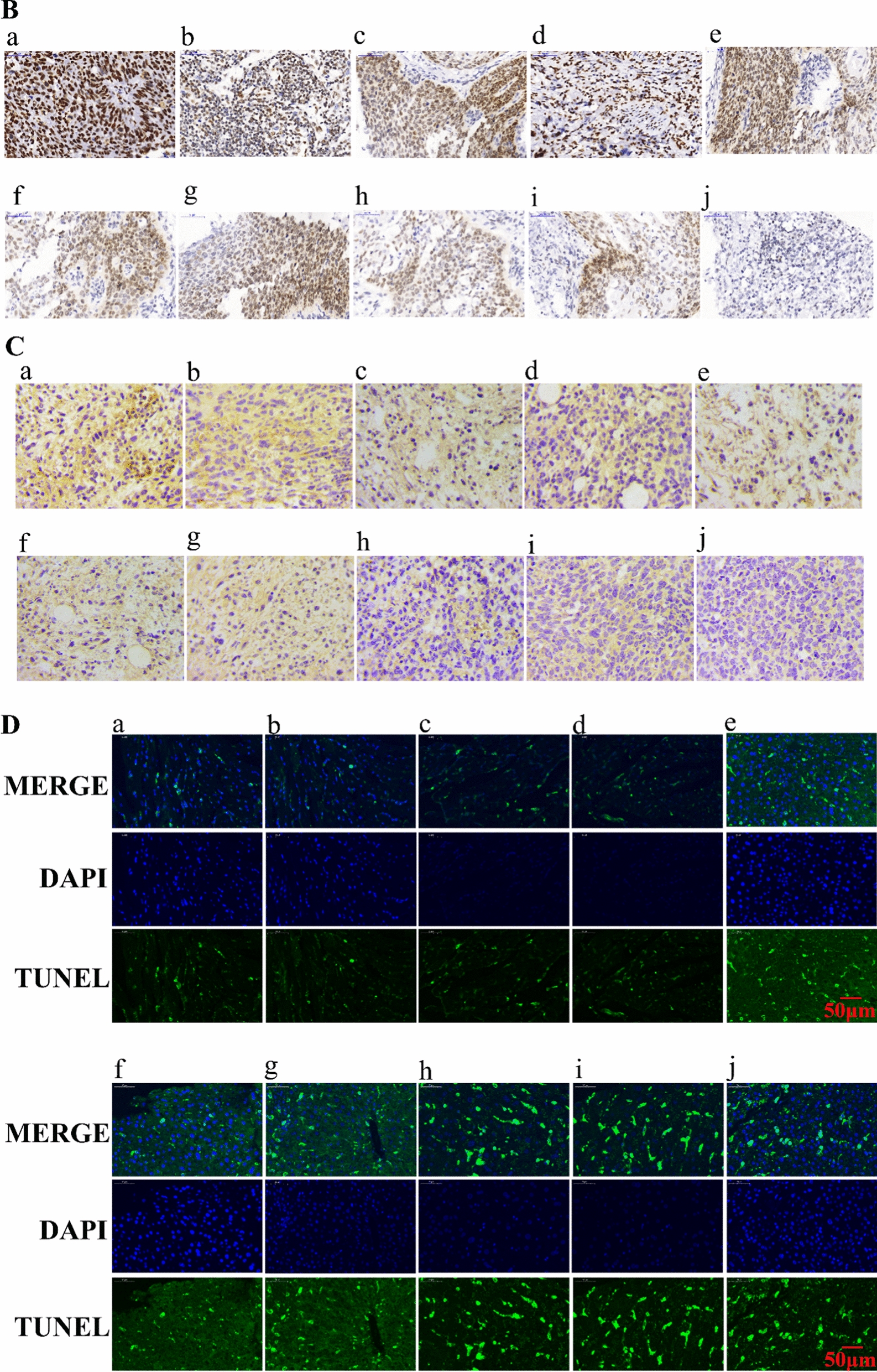
Sections of the tumor and main organs taken from mice of the saline group, solution groups and NLC groups (GA: 2 mg/kg, PTX: 1.7 mg/kg). HE staining of main organs (**A**) (Magnification 400×), Ki67 staining (**B**), CD31 staining (**C**) and TUNEL staining (**D**) of tumor sections after different treatments. (*a*: saline, *b*: Taxol, *c*: GA-Sol, *d*: PTX-Sol, *e*: GA/PTX-Sol, *f*: GA-NLC, *g*: PTX-NLC, *h*: GA/PTX-NLC, *i*: RGE-GA/PTX-NLC, and *j*: R9dGR-GA/PTX-NLC). Scale bar = 50 μm

The change of body weight with time of mice bearing tumor is one of the commonly used safety markers. The inhibition rate of body weight (IRBW %) of different groups were calculated. There was no notable changes were observed with in body weight in PTX-Sol, PTX-NLC, GA-NLC, GA/PTX-NLC, RGE-GA/PTX-NLC and R9dGR-GA/PTX-NLC groups during therapy period (Fig. 8e). However, the body weight of the mice subjected with Taxol, GA-Sol, and PTX/GA-Sol showed decreased with IRBW of 9.55%, 12.36%, 8.48% (Fig. 8f). The weight loss of the Taxol, GA-Sol, and PTX/GA-Sol groups were likely due to the nontargeted characteristics. As for Taxol, the formulations’ solvent system (cremophor) is also a vital factor for its toxicity. The RGE and R9dGR modified GA/PTX-NLC were well tolerated, suggesting that the potential off-target uptake of RGE-GA/PTX-NLC and R9dGR-GA/PTX-NLC by normal cells may not cause a detectable damage. At the same time, after the drug was encapsulated by NLC, the toxic and side effects were significantly reduced. The above results showed that the GA/PTX-NLC delivery system mediated by R9dGR was more efficient and safer than Taxol. The microscopic changes of organs of different treatment groups mice were observed histologically to assess the toxicity of different formulations. The results of HE were consistent with the above results (Fig. 9A). There were no significant histological change in the main organs in RGE and R9dGR modified NLCs group, indicating the targeted NLCs did not cause significantly systemic toxicity. Moreover, the visceral indexes of mice in targeted NLCs groups were calculated. There was no significant difference between the saline and targeted NLC groups in heart indexes, liver indexes, lung indexes, and kidney indexes; while the saline group showed splenomegaly (Additional file 1: Fig. S1F). The potential toxicity of different preparations was further evaluated by measuring the serum levels of AST, ALT, CRE, and CK [55]. According to the manufacturer’s protocol, serum ALT, AST, CRE, and CK were assayed with commercial diagnostic kits and a multifunctional microplate reader. Significantly elevated levels of each indicator indicate the presence of a corresponding toxicity. RGE-GA/PTX-NLC and R9dGR-GA/PTX-NLC reduced the serum AST and ALT activities (Fig. 8g), probably due to a hepatoprotective effect [56]. For CRE, there was no significant increase related to the conjugated formulations compare to the control group (Fig. 8g). In addition, there was no significant difference in serum CK level between the saline group and targeted NLC groups (Fig. 8g). Consequently, R9dGR-GA/PTX-NLC could significantly improve the efficacy and reduce the toxic side effects of GA/PTX in the treatment of tumors.

### Immunohistochemistry analysis

Immunohistochemical staining of Ki-67 was used to detect the proliferation of tumor cells in vivo. As displayed in Fig. 9B, R9dGR modified GA/PTX-NLC exhibited a significantly lower expression level of Ki67 in comparison with the other groups, namely, the inhibition effect of R9dGR-GA/PTX-NLC group was significantly obvious than other groups. The excellent antiproliferative capacity of R9dGR-GA/PTX-NLC was the result of the better recognition and penetration of tumor cells of R9dGR.

CD31 is the most sensitive and specific endothelial marker. Therefore, the anti-CD31 antibody was used to stain the blood vessels of 4T1 tumor to verify whether the anti-tumor effect of different preparations was related to the anti-angiogenesis effect. The results showed that the distribution of tumor blood vessels in mice of saline group was heterogeneous and tortuous. Regardless of the GA-NLC groups, GA/PTX-NLC, RGE-GA/PTX-NLC, R9dGR-GA/PTX-NLC groups, the tumor blood vessel was affected by different degrees. This is consistent with previous reports that GA could reduce blood vessel density in immunohistological studies related to CD31 [57]. As can be seen from Fig. 9C, RGE and R9dGR improved significantly antiangiogenic effect GA/PTX-NLC.

Specifically, the saline group only demonstrated close to no TUNEL positivity. In contrast, GA and/or PTX-treated groups exhibited TUNEL positivity. The treatment of GA combined with PTX exhibited more apoptotic cells than the single drug (Fig. 9D). The images also displayed that GA and PTX loaded by NLC could induce the apoptotic death of cancer cells to a greater extent than the treatment of the solution and Taxol with identical dose. It is worth noting that the tumor tissues of mice groups treated by RGE-GA/PTX-NLC and R9dGR-GA/PTX-NLC exhibited the most apoptotic cells in comparison with the tumor tissues of other mice groups. The trend of apoptotic analysis was consistent with the above results of in vivo anti-tumor effect.

### Pharmacokinetic study in mice

**Fig. 10:**
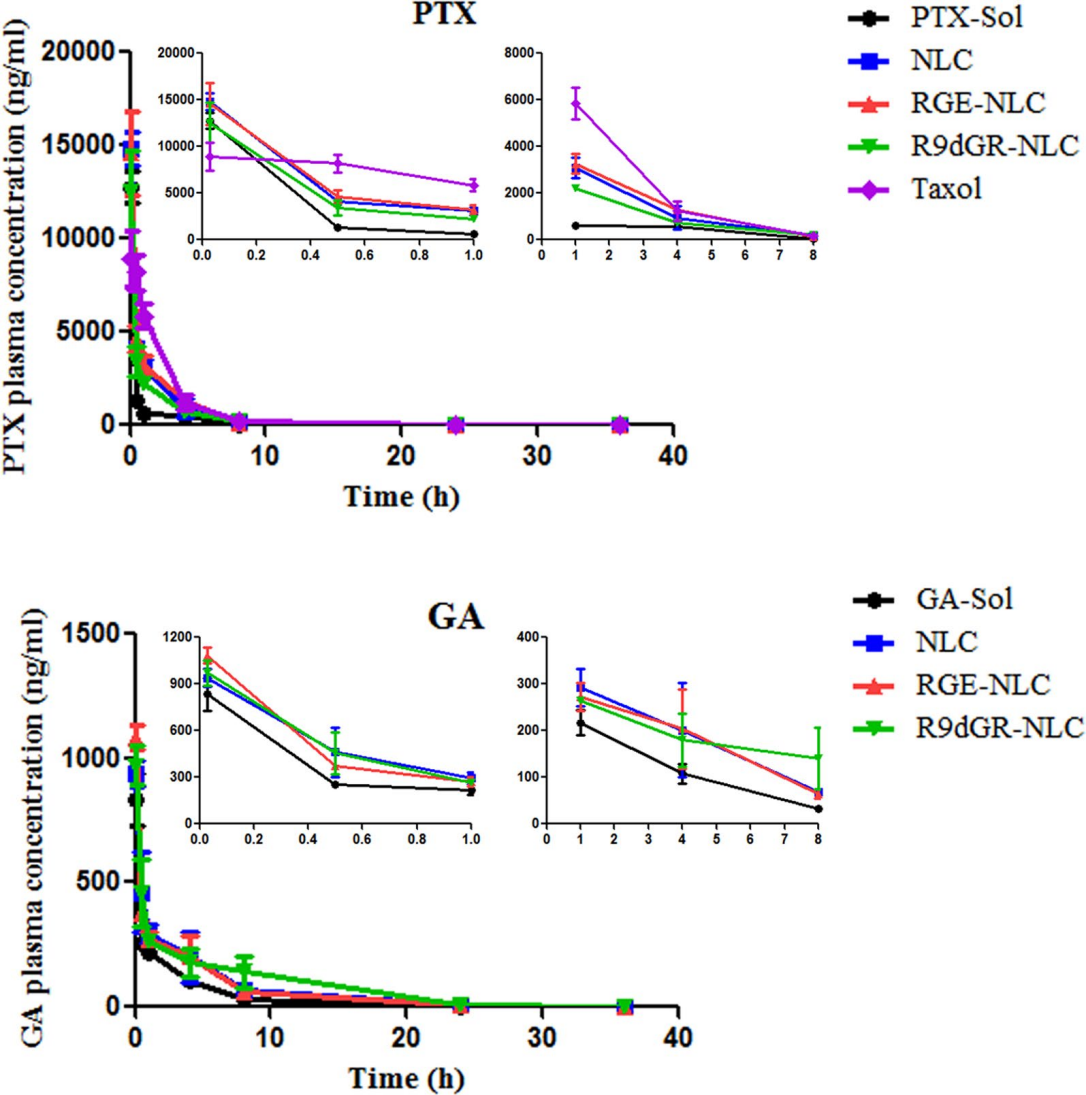
In vivo pharmacokinetic study of GA and PTX in mice (GA: 8 mg/kg, PTX: 6.8 mg/kg). Results are expressed as mean ± SD, n = 3

We determined in vivo GA and PTX concentration by UPLC-MS/MS. The pharmacokinetic curve of plasma concentrations of GA and PTX over time after intravenous injection was shown in Fig. 10. The following pharmacokinetic parameters: plasma elimination half-life (t_1/2_), area under the curve (AUC), mean residence time (MRT) and total body clearance (CL) [58] are listed in Table 3 and Table 4. The above parameters of GA and PTX were acquired using noncompartmental analysis of plasma concentration at the selected time point. The plasma concentrations of GA and PTX in GA/PTX-Sol were 33.02 and 58.78 ng/mL at 8 h, respectively, while the concentrations were 140.27 and 195.81 ng/mL at 8 h after intravenous injection of R9dGR-GA/PTX-NLC, respectively. The clearance (CL) of GA and PTX in GA/PTX-Sol group was higher than that of GA/PTX-NLC groups. For GA, t_1/2_, AUC, and MRT values of the R9dGR-GA/PTX-NLC group were 1.62-, 2.15-, and 1.56-fold higher than those of GA/PTX-Sol group, respectively; for PTX, t_1/2_, AUC, and MRT values of the R9dGR-GA/PTX-NLC group were 1.33-, 1.77-, and 1.29- fold higher than those of GA/PTX-Sol group, respectively. The results of pharmacokinetics showed that both the targeted and nontargeted NLCs had the characteristics of high stability and long circulation, which could make sure that GA and PTX were efficiently accumulated in tumor tissue. In addition, surface pegylation prevents plasma proteins from attaching to the NLCs surface and reduces the uptake of NLCs by the reticuloendothelial system. The plasma concentrations of GA and PTX in mice given GA/PTX-NLC were higher than those in mice given GA/PTX-Sol. Meanwhile, although the PTX in Taxol has a higher AUC and a lower CL than the PTX in GA/PTX-NLC, it may not demonstrate a better tumor therapeutic effect due to the lack of targeting.

**Table 3:** Pharmacokinetic parameters of PTX in different formulations

Parameter	Units	PTX-Sol	Taxol	GA/PTX-NLC	RGE-GA/PTX-NLC	R9dGR-GA/PTX-NLC
AUC_0→∞_	μg/mL h	7.66 ± 0.79	22.58 ± 3.55**	16.66 ± 3.35**	17.65 ± 1.83**	13.53 ± 1.83*
t½	h	2.62 ± 0.20	2.16 ± 0.16	3.25 ± 0.45**	2.92 ± 0.03	3.48 ± 0.10**
CL	mL/h/kg	960.32 ± 100.05	328.26 ± 47.80***	451.69 ± 101.70***	416.52 ± 43.27***	546.34 ± 75.85***
MRT	h	1.88 ± 0.14	1.92 ± 0.04	2.23 ± 0.32	2.11 ± 0.02	2.43 ± 0.09*

*P < 0.05, **P < 0.01, ***P < 0.001 compared with PTX-Sol. Results are expressed as mean ± SD (n = 3)

**Table 4:** Pharmacokinetic parameters of GA in different formulations

Parameter	Units	GA-Sol	GA/PTX-NLC	RGE-GA/PTX-NLC	R9dGR-GA/PTX-NLC
AUC_0→∞_	μg/mL h	1.44 ± 0.23	2.56 ± 0.61	2.45 ± 0.57	3.10 ± 0.99*
t½	h	2.70 ± 0.68	5.12 ± 0.75**	4.72 ± 0.60**	4.38 ± 0.33*
CL	mL/h/kg	5647.32 ± 963.53	3248.17 ± 758.95**	3381.66 ± 772.54*	2754.72 ± 824.02**
MRT	h	3.29 ± 0.24	4.54 ± 0.24**	4.37 ± 0.44**	5.14 ± 0.52***

*P < 0.05, **P < 0.01, ***P < 0.001 compared with GA-Sol. Results are expressed as mean ± SD (n = 3)

## Conclusion

In this research, the optimal ratio of GA and PTX was firstly investigated to achieve the maximal in vitro anticancer efficiency. Then, we successfully developed a R9dGR-modified GA/PTX-loaded NLC (R9dGR-GA/PTX-NLC). The multifunctional nanocarrier system as an exhaustive anti-cancer therapy exhibited a small size, high encapsulation efficiency, sustainable release, prolonged circulation time, a higher cytotoxicity against 4T1 cells in vitro, and better targeting and penetrability to tumor tissues and tumor cells; which resulted in stronger anti-tumor effect, increased tumor cell apoptosis, and lower systemic toxicity in 4T1 tumor model in mice. GA not only overcome the multidrug resistance of PTX by inhibiting P-gp activity in MCF-7/ADR cells, but also inhibited MDA-MB-231 cells migration and invasion in vitro, playing a crucial role in preventing and treating the lung metastasis of breast cancer caused by PTX; meanwhile, the synergistic anti-tumor effect of GA and PTX has also been verified in vitro and in vivo experiments. Therefore, we can conclude that R9dGR-GA/PTX-NLC as a nanoparticle-based multi-drug combination therapy is supposed to be a novel insight for targeted therapy of breast cancer, which could exert synergistic antitumor effect, overcome multidrug resistance, and suppress metastasis of cancer cell.

## Supplementary information

**Additional file 1: Fig. S1.** A: DSPE-PEG_2K_-COOH MALDI-TOF REPORT; B: RGERPPR MALDI-TOF REPORT; C: DSPE-PEG_2K_-RGERPPR MALDI-TOF REPORT; D: R9dGR(RRRRRRRRR-dGR) MALDI-TOF REPORT; E: DSPE-PEG_2K_-R9dGR (RRRRRRRRR-dGR) MALDI-TOF REPORT. F: the visceral indexes of mice in different groups. G: Particle size of GA/PTX-NLC, RGE-GA/PTX-NLC and R9dGR-GA/PTX-NLC (by TEM images). H: R9dGR-GA/PTX-NLC. **Fig. S2**. The microscopy image of cellular uptake in MDA-MB-231 (A), 4T1 (B), and MCF-7 (C) cells after treatment with Cou-6-Sol, Cou-6-loaded targeted and nontargeted NLC (scale bar = 100 μm)

## Data Availability

The datasets used and/or analyzed during the current study are available from the corresponding author on reasonable request.

## Abbreviations

AST: Aspartate aminotransferase
ALT: Alanine aminotransferase
CPP: Cell penetrating peptide
CIR: Cell inhibition rate
CRE: Creatinine
CI: Combination index
Cou-6: Coumarin-6
CK: Creatine kinase
CCK-8: Cell Counting Kit 8
DL: Drug loading capacity
Dir: 1,10-Dioctadecyl-3,3,3,3-tetramethyl indotricarbocyanine iodide
EE: Encapsulation efficacy
GA: Gambogic acid
HSRs: Hypersensitivity reactions
IVIS: In-Vivo Imaging System
IRBW: The inhibition rate of body weight
MDR: Multidrug resistance
NRP-1: Neuropilin-1
NLCs: Nanostructured lipid carriers
PTX: Paclitaxel
PDI: Polydispersity index
P-gp: P-glycoprotein
R9dGR: RRRRRRRRRdGR
RGE: RGERPPR
RBCs: Red blood cells
Rh-123: Rhodamine 123
RPM: Revolutions per minute
SLN: Solid lipid nanoparticle
TCMs: Traditional Chinese medicines
TMEM: Tumor microenvironment of metastasis
TIR: Tumor inhibition ratio
TGI: Tumor growth inhibition
UPLC: Ultra performance liquid chromatography
