# Supplementary material for: Dual drug-loaded nano-platform for targeted cancer therapy: toward clinical therapeutic efficacy of multifunctionality
Source: J Nanobiotechnology. 2020 Sep 4;18:123. doi: 10.1186/s12951-020-00681-8 (PMC7650261; doi:10.1186/s12951-020-00681-8)
Supplement: Supplementary file 1 — Additional file 1: Fig. S1. A: DSPE-PEG2K-COOH MALDI-TOF REPORT; B: RGERPPR MALDI-TOF REPORT; C: DSPE-PEG2K-RGERPPR MALDI-TOF REPORT; D: R9dGR(RRRRRRRRR-dGR) MALDI-TOF REPORT; E: DSPE-PEG2K-R9dGR (RRRRRRRRR-dGR) MALDI-TOF REPORT. F: the visceral indexes of mice in different groups. G: Particle size of GA/PTX-NLC, RGE-GA/PTX-NLC and R9dGR-GA/PTX-NLC (by TEM images). H: R9dGR-GA/PTX-NLC. Fig. S2. The microscopy image of cellular uptake in MDA-MB-231 (A), 4T1 (B), and MCF-7 (C) cells after treatment with Cou-6-Sol, Cou-6-loaded targeted and nontargeted NLC (scale bar = 100 μm) [file 12951_2020_681_MOESM1_ESM.doc]

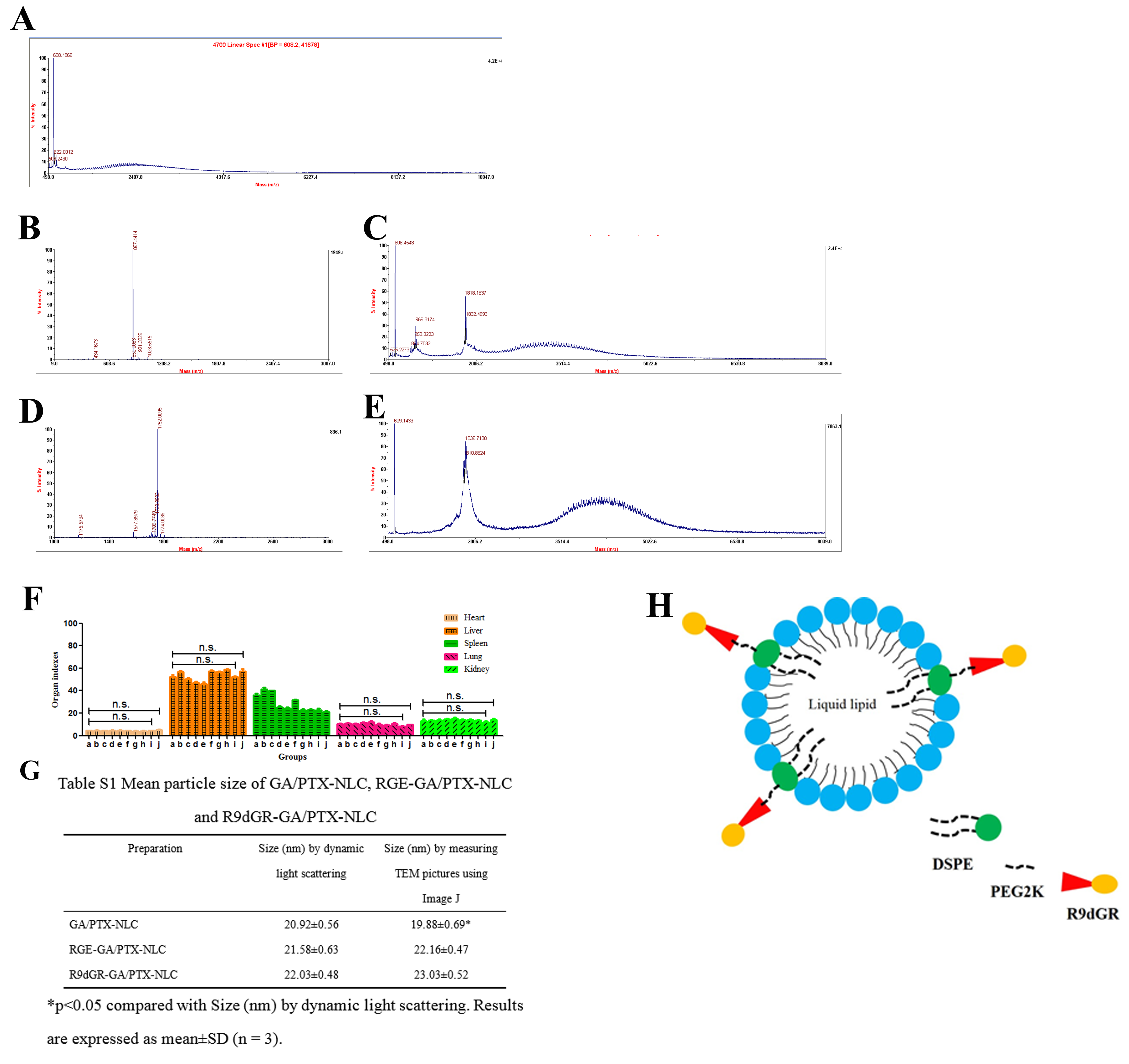


Figure S1 A: DSPE-PEG2K-COOH MALDI-TOF REPORT; B: RGERPPR MALDI-TOF REPORT; C: DSPE-PEG2K-RGERPPR MALDI-TOF REPORT; D: R9dGR(RRRRRRRRR-dGR) MALDI-TOF REPORT; E: DSPE-PEG2K-R9dGR (RRRRRRRRR-dGR) MALDI-TOF REPORT. F: the visceral indexes of mice in different groups. G: Particle size of GA/PTX-NLC, RGE-GA/PTX-NLC and R9dGR-GA/PTX-NLC (by TEM images). H: R9dGR-GA/PTX-NLC.


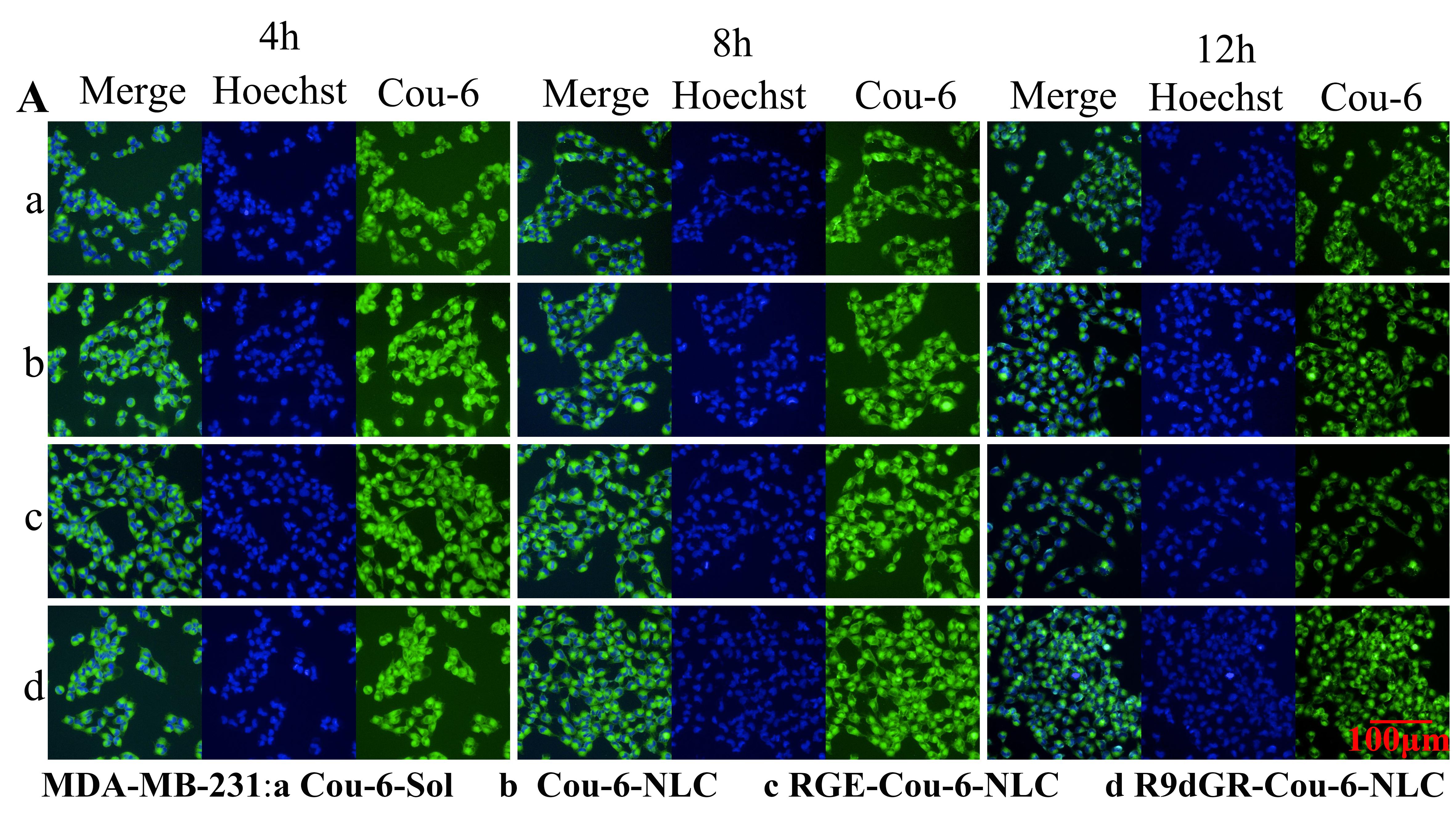


Figure S2 The microscopy image of cellular uptake in MDA-MB-231 (A), 4T1 (B), and MCF-7 (C) cells after treatment with Cou-6-Sol, Cou-6-loaded targeted and nontargeted NLC (scale bar=100 μm).
